# SIRT3, a metabolic target linked to ataxia-telangiectasia mutated (ATM) gene deficiency in diffuse large B-cell lymphoma

**DOI:** 10.1038/s41598-020-78193-6

**Published:** 2020-12-03

**Authors:** Kavita Bhalla, Sausan Jaber, Kayla Reagan, Arielle Hamburg, Karen F. Underwood, Aditya Jhajharia, Maninder Singh, Binny Bhandary, Shambhu Bhat, Nahid M. Nanaji, Ruching Hisa, Carrie McCracken, Heather Huot Creasy, Rena G. Lapidus, Tami Kingsbury, Dirk Mayer, Brian Polster, Ronald B. Gartenhaus

**Affiliations:** 1 Marlene and Stewart Greenebaum Comprehensive Cancer Center, Department of Medicine, University of Maryland School of Medicine, Baltimore, MD 21201 USA; 2 Department of Anesthesiology, University of Maryland, Baltimore, MD 21201 USA; 3 Department of Diagnostic Radiology and Nuclear Medicine, University of Maryland, Baltimore, MD USA; 4 Veterans Administration Medical Center, Baltimore, MD 21201 USA; 5 Electron Microscopy Core Imaging Facility, Department of Medicine, University of Maryland, Baltimore, USA; 6 Institute of Genome Sciences, University of Maryland, Baltimore, MD 21201 USA; 7 Department of Physiology, The Center for Stem Cell Biology and Regenerative Medicine, Baltimore, MD 21201 USA; 8 Hunter Holmes McGuire Veterans Administration Medical Center, Virginia Commonwealth University, School of Medicine, Richmond, VA USA

**Keywords:** Lymphoma, B-cell lymphoma

## Abstract

Inactivation of Ataxia-telangiectasia mutated (ATM) gene results in an increased risk to develop cancer. We show that ATM deficiency in diffuse large B-cell lymphoma (DLBCL) significantly induce mitochondrial deacetylase sirtuin-3 (SIRT3) activity, disrupted mitochondrial structure, decreased mitochondrial respiration, and compromised TCA flux compared with DLBCL cells expressing wild type (WT)-ATM. This corresponded to enrichment of glutamate receptor and glutamine pathways in ATM deficient background compared to WT-ATM DLBCL cells. ATM^−/−^ DLBCL cells have decreased apoptosis in contrast to radiosensitive non-cancerous A-T cells. In vivo studies using gain and loss of SIRT3 expression showed that SIRT3 promotes growth of ATM CRISPR knockout DLBCL xenografts compared to wild-type ATM control xenografts. Importantly, screening of DLBCL patient samples identified SIRT3 as a putative therapeutic target, and validated an inverse relationship between ATM and SIRT3 expression. Our data predicts SIRT3 as an important therapeutic target for DLBCL patients with ATM null phenotype.

## Introduction

Patients with defects in A-T mutated gene (*ATM*) develop a progressive cerebellar degeneration leading to severe neuromotor dysfunction^1^. ATM kinase is a master regulator of DNA damage response (DDR)^1^. A-T patients carry 86% of truncating mutations and 14–26% of dominant negative missense mutations^2,3^. Loss of ATM protein results in genetic instability and altered DNA repair^2,3^. Consequently, A-T patients are at increased risk of developing cancer^3^. Clinical evidence indicates that 30–40% of A-T patients develop lymphoid malignancies^2,4^. Further, ATM^−/−^ mice was shown to develop lymphoma that resembles human diffuse large B-cell lymphoma (DLBCL)^4,5^. ATM mutations have been found to be associated with DLBCL^6^, which represents most common subtype of lymphoma worldwide^7^. Almost 50% of DLBCL patients develop relapsed/resistant disease with limited therapeutic options, leading to a lower survival rate^8^. Additionally, it is a major challenge to effectively treat refractory/relapsed cases due in part to the intrinsic tumor heterogeneity. Based on RNA profile of cell of origin (COO), DLBCL is most often classified as germinal center B cells (GCB) or activated B cell like (ABC). Based on transcriptional profiling, DLBCL can be classified as B-cell receptor (BCR)-DLBCL subtype or Oxidative phosphorylation subset (Oxphos-DLBCL)^9^. BCR tumors are highly glycolytic, whereas, Oxphos-DLBCL tumors depict an increased expression of Oxphos genes. To add to the complexity of phenotype, DLBCL patients fall into different prognostic categories; patients with GCB molecular signature have improved overall survival (60–70%) compared with that of patients with ABC signature (30–40%)^10^. Therefore, unraveling lymphoma-specific metabolic pathways occurring in ATM deficient background will provide critical mechanistic insights leading towards effective therapies.

Previously published studies suggest that B-cell malignancies with ATM null phenotype have poor treatment response and are refractory to conventional therapies and DNA damaging agents. Hence, there is a need for therapeutic strategies that act independent of DDR that can target ATM deficient lymphoma and other related B-cell malignancies in ATM deficient background. It is becoming evident that ATM orchestrates several metabolic pathways beyond sensing DNA damage^11^. For example activation of nuclear respiratory factor 1 (NRF1) pathway to maintain cellular homeostasis^12^. Another report showed that nuclear respiratory factor 2 (NRF2) pathway can be exploited for therapeutic targeting of chronic lymphocytic leukemia (CLL) with ATM null phenotype^13^. Therapeutic targeting of metabolic pathways that function independent of DDR to support tumor survival represents an attractive strategy for improving treatment efficiency in ATM deficient lymphoid malignancies.

Mitochondrial metabolism is a central checkpoint for cancer development. We recently reported that lymphoma cells adjust their metabolic demand by modulating mitochondrial function under hypoxic stress^14^. ATM deficiency is associated with mitochondrial dysfunction^15^. Alterations in TCA metabolites were reported in the cerebellum of ATM^−/−^ mice^16^. ATM regulates utilization of the pentose-phosphate pathway (PPP) during senescence. ATM is activated in response to oxidative stress in mitochondria in the absence of DDR^17^, and also plays a role in regulating reactive oxygen species (ROS)^18^. Oxidative stress in A-T neuronal cells is likely a result of diminished mitochondrial function^19^. In order to prevent ROS induced cell death, cancer cells use various protective mechanisms including activation of ROS-detoxifying enzymes, (NAD+ dependent class III histone deacetylase) SIRT1 and SIRT3^20^. These NAD+ dependent histone deacetylases are important for maintaining mitochondrial integrity and cancer progression^21–24^. We recently showed that human antigen-R (HuR) associated mRNAs are regulated in an ATM-dependent manner including the Forkhead Box protein, FOXO3^25^. HuR also alters expression of SIRT1^26^, which plays an important role in DNA damage repair and maintenance of genomic integrity^27^. Our published data support defining the role of altered sirtuin signaling in ATM-deficient lymphoma. Based on these findings, we embarked on interrogating the role of FOXO-SIRT axis in lymphoma development in the context of ATM deficiency. We show that SRIT3 is new therapeutic target in DLBCL with ATM null phenotype.

## Results

### Effect of ATM deficiency on FOXO3A- SIRT1 axis in DLBCL

We earlier published that expression of FOXO3a is regulated in ATM dependent manner in normal B-lymphocytes. We observed that association of RNA binding protein HuR to FOXO3a was dependent on ATM activity^25^. Another report showed that HuR regulates SIRT1 expression in human cervical carcinoma HeLa cells^26^. Therefore, we first investigated if ATM deficiency impacts expression of these oncogenic proteins FOXO3A and SIRT1 in DLBCL. We inhibited ATM expression in DLBCL cells lines representing different subsets of DLBCL: HLY, SUDHL2 (ABC subtype) and SUDHL6 (GCB subtype) (Supplementary Fig.S1A). There was a modest decrease in RNA and protein expression of FOXO3A in shATM DLBCL cells as compared with non-target (NT) control (Supplementary Fig. S1B,D). No difference was observed in total SIRT1 expression in ATM inhibited DLBCL cells (Supplementary Fig. S1B,D). Although, immunofluorescence staining of SIRT1 showed inhibition in nuclear SIRT1 expression in ATM inhibited DLBCL cells compared with ATM-WT DLBCL cells, this difference was not significantly different from that observed in ATM deficient normal fibroblasts when compared to WT-ATM cells (Supplementary Fig. S1E). These observations suggest that ATM deficiency does not significantly impact FOXO3a/SIRT1 expression in DLBCL.

### Stimulation of SIRT3 expression in ATM-deficient DLBCL

**Figure 1:**
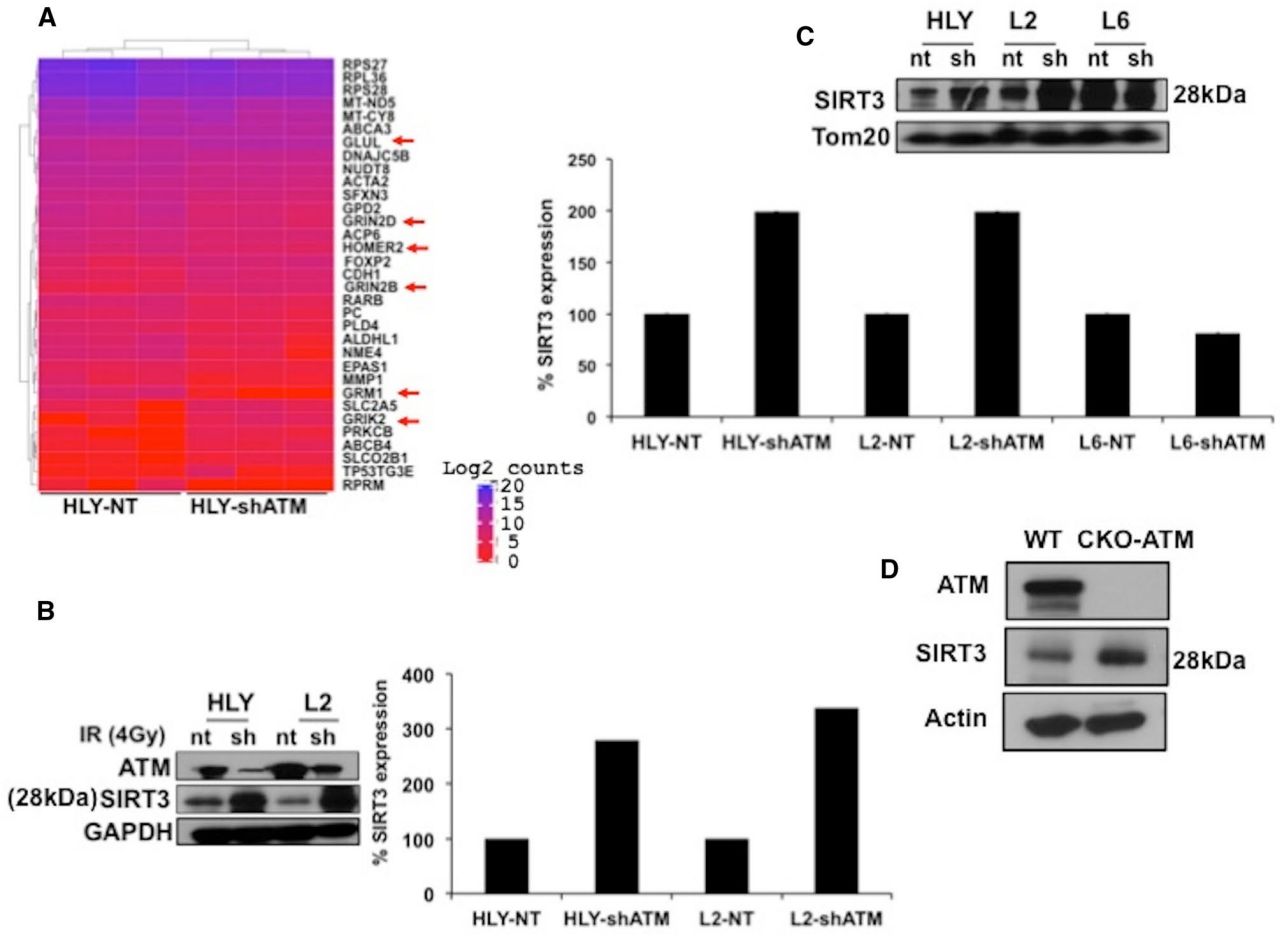
Regulation in SIRT3 axis in response to ATM deficiency in DLBCL. (**A**) Gene clustering illustrated by a heatmap of gene expression across samples (n = 3/group) indicate glutamate receptor and glutamine synthesis gene targets as top regulated pathways in shATM cell line compared to its non-target control (NT). Genes associated with top regulated pathways identified by IPA are indicated in heat map by red arrows. Gene expression values come from log2 raw counts generated by htseq-count. Heat map was created using the R library Complex Heatmap, which is part of Bioconductor software (3.1): http://bioconductor.org/packages/release/bioc/html/ComplexHeatmap.html. (**B**,**C**) Western blot analysis and quantitation of SIRT3 expression from fractionated cellular extracts (**B**) from total cell lysate (**C**) from mitochondrial cell lysates obtained from ABC-DLBCL cell lines HLY and SUDHL2 (L2) and GCB-DLBCL cell lines SUDHL6 (L6). Quantitation of SIRT3 protein expression is depicted below. The western analysis data and bar chart illustrating relative quantitation presented in (**B**) and (**C**) are the representative experiment of three independent experiments that were initiated from independent cell culture preparations. (**D**) SIRT3 expression in CRISPR-knockout (CKO) ATM DLBCL cell line, HLY. Size of mitochondrial isoform of SIRT3 (28 kDa) is depicted. The expression level of SIRT3 in CKO-ATM DLBCL cell line as determined by western blot analysis depicted here is a representative experiment of three independent experiments.

Next, to systematically identify metabolic pathways that contribute specifically to DLBCL growth in ATM deficient background, we performed global RNA sequencing. We previously showed that ATM controls expression of critical oncogenic proteins posttranscriptionally^25^. Therefore, polysomal RNA was isolated from ABC-DLBCL cell line HLY expressing WT-ATM and sh-ATM lentivirus and RNA sequence analysis was done to identify metabolic targets that are post transcriptionally regulated by ATM deficiency. Several metabolic pathways, including; glucose metabolism, electron transport chain, oxidative phosphorylation, oxidative stress, ribosomal proteins, ROS, mitochondrial function and glutamine biosynthesis were altered in background of ATM deficiency, as identified by IPA analysis, and were included in the heat map. Interestingly, some of the top pathways regulated included glutamate receptor and glutamine signaling, which are targets of SIRT3 (Fig. 1A). RNA sequencing analysis highlighted the influence of SIRT3 dependent pathways in development of DLBCL. Therefore, to validate RNA sequencing data, we examined expression of SIRT3 in ATM inhibited DLBCL cell lines. Full length human SIRT3 (~ 44 kDa) is encoded in the nucleus. During cellular stress, the longer enzymatically inactive form is imported to mitochondrial matrix by a canonical mitochondrial targeting sequence where it cleaves the N-terminal leading to an active (28 kDa) isoform of SIRT3 that resides in mitochondria^28–30^. Strikingly, expression for active isoform of SIRT3 was enhanced in ABC-DLBCL cell lines that were inhibited for ATM expression compared to NT expressing DLBCL cell lines (Fig. 1B). We subsequently, performed chemical fractionation to measure expression of mitochondrial SIRT3, which was significantly induced in ABC-DLBCL compared to wild type (WT)-ATM expressing NT-DLBCL cells (Fig. 1C). No difference in SIRT3 expression was observed in SUDHL6-DLBCL cell line (GCB-DLBCL subtype) expressing NT and shATM lentivirus (Fig. 1C). We independently confirmed stimulation of mitochondrial SIRT3 expression in another ABC-DLBCL cell line U2932 (S2A). ATM inhibition did not effect expression of 44-kDa long inactive (nuclear) isoform of SIRT3 in DLBCL cell lines (Supplementary Fig. S2B). We also generated CRISPR knockout (CKO) of ATM in DLBCL cell line HLY. The status of DLBCL cell lines for WT-ATM and mutation in ATM was confirmed by DNA sequencing. ATM deficiency significantly induced SIRT3 expression compared to HLY cell line expressing WT-ATM (Fig. 1D). Importantly, ATM deficiency did not regulate mitochondrial SIRT3 expression in normal ATM^−/−^ (GM03332) cells compared with ATM^+/+^GM02184 (Supplementary Fig. S2C). To validate findings of RNA sequencing analysis, we also confirmed utilization of glutamate, a metabolic precursor of gamma amino butyric acid (GABA). Interestingly, increased utilization of GABA was seen in ATM inhibited DLBCL cells (Supplementary Fig. S2D). Together, these results indicate that SIRT3 stimulation is specific to ABC-DLBCL in ATM deficient background sparing normal ATM^−/−^ cells.

### Stimulation of SIRT3 function in ATM-deficient DLBCL

**Figure 2:**
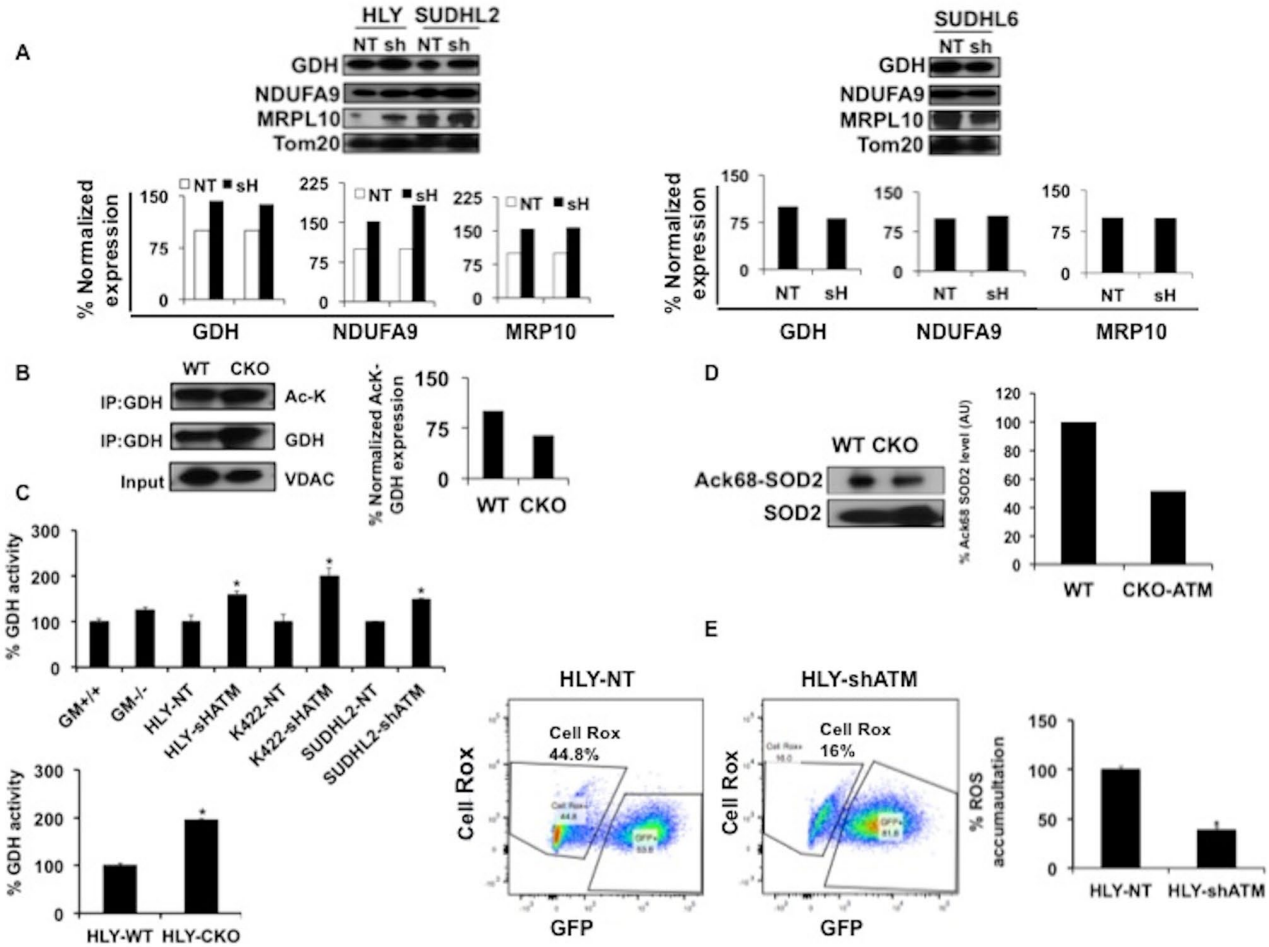
ATM inhibition stimulated SIRT3 activity in DLBCL. (**A**) Expression of SIRT3 targets in DLBCL cell lines inhibited for ATM expression compared to non-target controls. Expression of these targets in ABC DLBCL cell lines (HLY AND SUDHL2) and GCB cell line (SUDHL6) is depicted and quantitated normalized expression is provided below. Protein expression was quantitated using Image J software. Protein expression in shATM-DLBCL cell lines is expressed as relative percentage expression compared to non-target control. Protein abundance data shown here is a representative from triplicate experiments that were initiated from independent cell cultures. (**B**) Effects of SIRT3 on GDH acetylation in ATM-WT and ATM deficient DLBCL cell line HLY. Western blotting was performed using AcK and GDH antibodies on immunoprecipitated (IP) GDH. Protein expression was quantitated using Image J software. Percentage of GDH acetylation normalized to total GDH expression is depicted. VDAC was used as input control loading. The images are representative of two independent experiments. All western blots were run under same experimental conditions. **(C)** GDH activity assay in GM control cells and DLBCL cell lines expressing nt-shRNA and ATM-shRNA, experiments were repeated three times and data are expressed as mean + SD, asterisks define significant difference *p < 0.05. (**D**) Percentage acetylation of SOD2 as determined by Ack-68-SOD2 antibody in ATM CRISPR knock out (CKO) DLBCL cells compared to WT-ATM. The percentage decrease in acetylated SOD2 expression in ATM-CKO cells was estimated by normalizing the acetylated expression levels to total SOD2 levels in both wild type-ATM and ATM deficient DLBCL groups. The images are representative of two independent experiments. All western blots were run under same experimental conditions. (**E**) Representative density blots of FACs analysis showing percentage ROS accumulation in DLBCL transduced with GFP tagged lentiviral particles expressing nt-shRNA and ATM-shRNA. Experiment was repeated n = 3, data are expressed as mean + SD, asterisks define significant difference p < 0.05.

We subsequently analyzed expression and activity of SIRT3 targets important for maintaining mitochondrial homeostasis: glutamate dehydrogenase (GDH), complex 1 subunit (NDUFA9) and Mitochondrial ribosomal protein (MRP10). Protein expression pattern revealed a trend towards increase in SIRT3 targets in ATM inhibited ABC-DLBCL cell lines HLY and SUDHL2 compared to WT-ATM expressing DLBCL cells (Fig. 2A). No difference in expression of SIRT3 targets was observed in SUDHL6-DLBCL cell line (GCB-DLBCL subtype) expressing NT and shATM lentivirus (Fig. 2A). We independently confirmed expression of GDH, NDUFA9 and MRP10 in additional ABC-DLBCL cell line U2932 that was inhibited for ATM expression. Similar to what we observed in ABC-DLBCL cell lines HLY and SUDHL2, we noticed that inhibition of ATM resulted in increased expression of SIRT3 targets in DLBCL cell line U2932 (Supplementary Fig. S3A). ATM inhibition in DLBCL cell lines did not effect expression of another SIRT3 target, AceCS2 (Acetyl-CoA synthetase 2) (Supplementary Fig. S3B). We next measured activity of GDH by determining acetylation of GDH-lysine (K9) by immunoprecipitation. Normalization of Ac-k9 expression with total GDH expression indicated a trend towards decrease in Ack9-GDH expression in absence of active ATM signaling compared to WT-ATM DLBCL (Fig. 2B), indicating increased GDH activity. We then independently confirmed increased GDH enzyme activity in ATM^−/−^ DLBCL cell lines compared to cells expressing WT-ATM using GDH enzyme assay activity kit (Fig. 2C). We also measured acetylation of another important SIRT3 target, superoxide dismutase (SOD2). SIRT3 dependent deacetylation of SOD2 residue (K68) results in increased SOD2 activity^31^. We observed a trend towards decrease in Lysine 68 acetylation of SOD2 when ATM was inhibited compared to WT-ATM DLBCL cells (Fig. 2D). We also noticed significant reduction of cellular ROS levels in ATM inhibited HLY cells compared to HLY cells expressing WT-ATM (Fig. 2E), including in the presence of genotoxic insult (Supplementary Fig. S3C). These results confirm that ATM deficiency results in stimulation of SIRT3 activity in DLBCL cells compared to DLBCL cells expressing WT-ATM.

### Effect of ATM inhibition on Mitochondrial Structure and function in DLBCL

**Figure 3:**
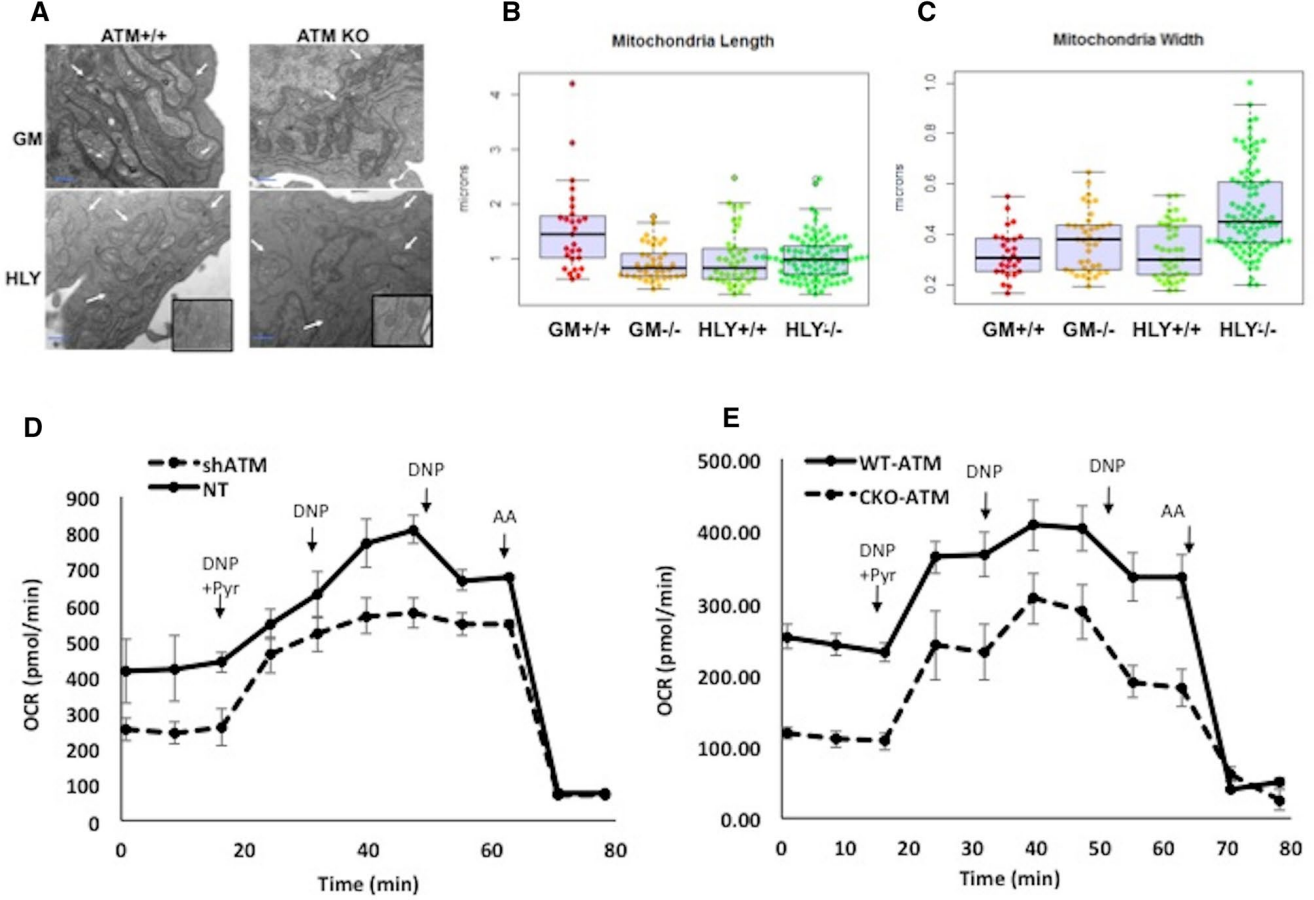
Electron microscopy and oxygen consumption rate (OCR) in ATM^+/+^ and ATM^−/−^ normal B-cell (GM) and DLBCL (HLY) cells. (**A**) Representative electron microscopy (EM) images of mitochondrial structure in ATM^+/+^ (GM02184), ATM^−/−^ (GM03332) and DLBCL cell lines, HLY-NT and shATM-HLY. Arrows pointing out mitochondria are shown in respective groups. Three replicates were used in respective groups for EM imaging. (**B**) Mitochondria length in GM control and DLBCL cell lines modified for ATM expression. Length of mitochondria in GM 02184 cells with wild type ATM^+/+^ was significantly more compared to mitochondrial length in ATM^−/−^ GM03332 and malignant HLY cells both with WT-ATM and ATM^−/−^, n = 3, p < 0.01. (**C**) Mitochondria width in GM control and DLBCL cell lines modified for ATM expression. Each dot represents one mitochondrion and crossbars represent mean + SD. No significant difference was observed in mitochondrial width between ATM^+/+^ GM 02184, ATM^−/−^ GM03332 and malignant ATM-WT HLY cells. The mitochondria in ATM^−/−^ HLY group were significantly wider in shape compared to mitochondria in normal ATM^+/+^ GM 02184 cells, p < 0.01. (**D**,**E**) Representative OCR traces from the DLBCL cell line HLY. Cells were genetically inhibited for ATM signaling using (**D**) lentiviral approach or (**E**) CRISPR. OCR was measured during the sequential addition of uncoupler DNP plus 10 mM pyruvate (Pyr), DNP alone twice, and finally an inhibitor of respiration, antimycin A, using Seahorse Extracellular Flux Analyzer. Inhibition of ATM decreased respiration rate in ATM^−/−^HLY cells compared with WT-ATM-HLY cells. Data are derived from n = 3 passages per cell line. Each experiment was set using n = 5–6 replicates per ATM^+/+^ and ATM^−/−^ cells. Graphs are represented as mean + SD of three independent experiments.

Although previous reports showed that ATM deficiency leads to mitochondrial abnormalities in normal lymphoblastoid cells and thymocytes^15,19^, it is unclear how ATM deficiency affects mitochondrial structure and function in lymphoma. Electron microscopy (EM) studies were performed to determine if ATM is required to maintain mitochondrial structural integrity in DLBCL. Mitochondria were found to be typical bacillus shaped in normal B-cells (GM02184) that express functional ATM (Fig. 3A). Similar to previous reports in A-T neuronal cells^15,19^; we found that mitochondria in the ATM^−/−^ normal (GM03332) cells were more rounded in shape (Fig. 3A). EM studies demonstrated that ATM-WT malignant B-cells consisted of mixed population of tubular and smaller mitochondria (Fig. 3A). Mitochondria from genetically inhibited ATM-DLBCL cells displayed greater variation in width and were found to be more swollen and round in shape with fragmented outer membrane and cristae (Fig. 3A). Measurement of mitochondrial length and width across different focal fields confirmed swollen mitochondrial structure in ATM-mutated DLBCL cells compared with wild-type ATM expressing cells (Fig. 3B,C). We also assessed expression of key proteins involved in mitochondrial fusion and fission to confirm observed structural variations. While Optic atrophy (OPA1) expression was reduced in sh-ATM DLBCL cells (Supplementary Fig. S4A), expression of, DRP1 (dynamin-related protein), was increased in ATM deficient DLBCL cell lines compared to WT-ATM cells (Supplementary Fig. S4B). These results, in combination with the observed mitochondrial structural changes are consistent with the possibility of increased mitochondrial fission in ATM^−/−^ DLBCL cells. Perhaps, mitochondrial fragmentation might be creating small swollen mitochondria in ATM^−/−^ DLBCL cells to regulate mitochondrial biogenesis. It has been suggested earlier that during stress mitochondrial network inhibit fusion of mitochondria to segregate damaged mitochondria from healthy by fission for controlling mitochondrial quality^32,33^. Structural changes in mitochondria are crucial to determine cell function and fate. Apparently, cancer cells regulate mitochondrial morphology on basis of their bioenergetics needs^34^. To further quantitate mitochondrial morphology, we calculated mitochondrial interconnectivity as published earlier^35,36^. We did not observe difference in mitochondrial interconnectivity in ATM deficient DLBCL cells as compared to the wild type ATM DLBCL cells. ATM deficiency has also been associated to increased mitochondrial mass in thymus to regulate mitochondrial homeostasis^15,37^. Therefore, we next investigated if ATM deficiency leads to changes in mitochondrial mass in DLBCL cells. We stained ATM-wild type and ATM-CKO cells using Mito Tracker green. Mitochondrial mass as estimated by flow cytometry depicted a two-fold increase in mitochondrial mass in HLY-CKO cells compared to ATM-WT HLY cells (Supplementary Fig. S4C). Mitochondrial structural abnormalities were accompanied by increased oxygen consumption rate (OCR) in *ATM*^−/−^ thymocytes^15^. However, in contrast, in ATM- deficient lymphoblastoid cells there was diminished mitochondrial respiration compared to wild type cells^19^. We observed a trend towards a reduced overall respiration rate in ATM^−/−^DLBCL cells compared with cells expressing WT-ATM (Fig. 3D,E). Quantification of this data revealed a 40–50% decrease in basal respiration rates, and maximal respiration was decreased by 25–30% in ATM^−/−^ DLBCL cells lines. The respiration rate for ATM-WT cells was set to 100% (Supplementary Fig. S4D). Our data indicates that active ATM signaling is indeed necessary for mitochondrial structural integrity in cancer cells similar to normal physiological conditions and SIRT3 stimulation does not alleviate structural changes in mitochondria in ATM deficient DLBCL cells. However, stimulation in SIRT3 helps DLBCL cells to maintain low respiratory requirements and contributes to regulate energy expenditure during metabolic stress in presence of ATM deficiency.

### Effect of ATM deficiency on TCA activity in DLBCL

**Figure 4:**
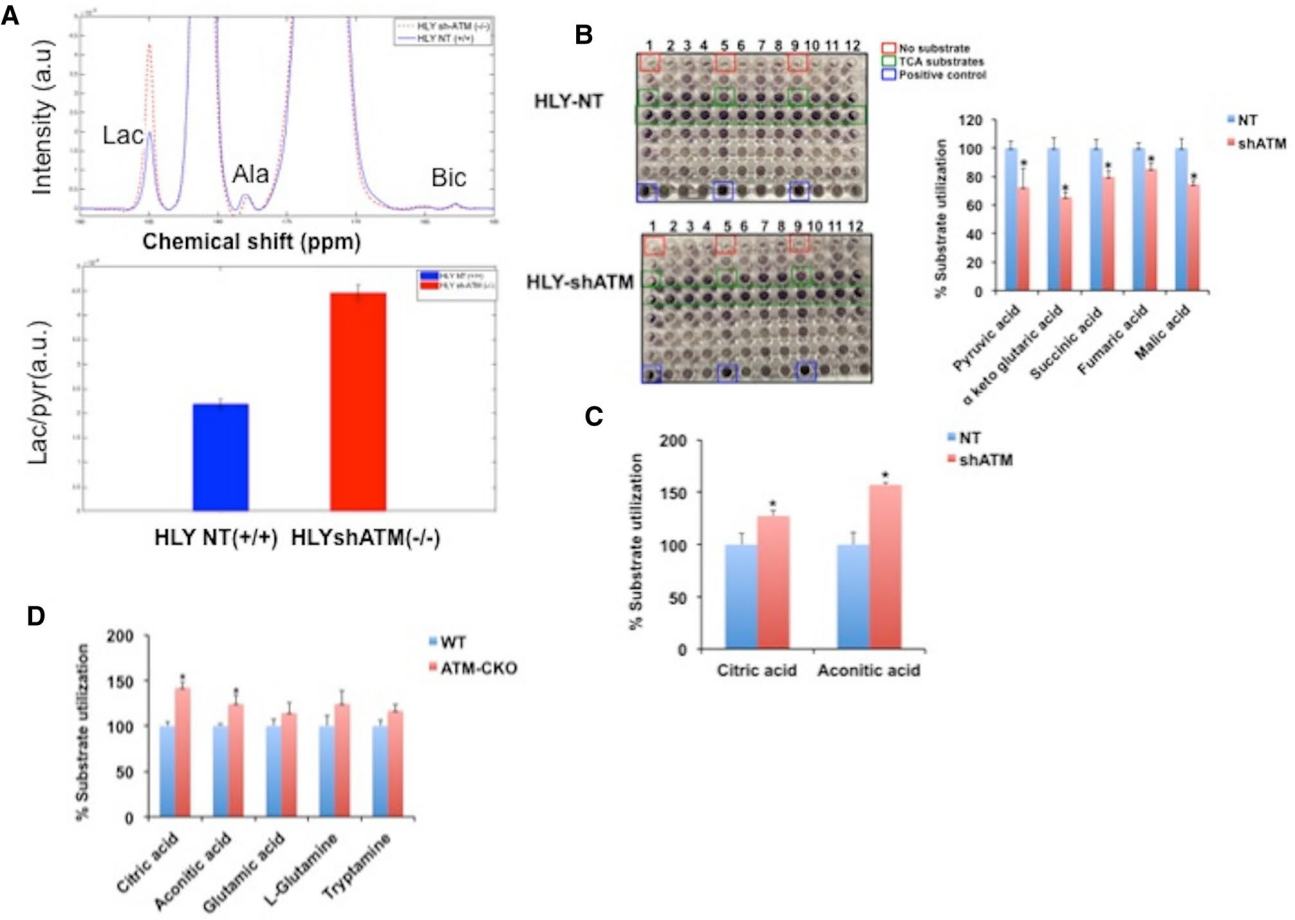
Metabolic measurements in DLBCL cells using hyperpolarized [1-C] pyruvate and BiOLOG assay. (**A**) The top panel represents the spectra chart for hyperpolarized C signal at ambient temperature after dissolution. Blue and red spectra correspond to metabolic process in the presence of HLY-NT (^+/+^) and HLY SH (^−/−^). Both spectra are normalized with respect to maximum pyruvate signal. HLY sh-ATM (^−/−^) shows higher conversion of pyruvate into lactate. In the spectra pyruvate, lactate, pyruvate hydrate, alanine and bicarbonate signals are at 172.8, 184.9, 181, 178.4, and 162.7 ppm, respectively. Bottom panel represents Lactate-to-pyruvate ratios for HLY NT (^+/+^) and HLY sh-ATM (^−/−^) cell lines in blue and red, respectively (n = 3 for each cell line). Experiment was repeated three times, data are expressed as mean + SE. (**B**) Suspension of DLBCL cells were seeded on a 96 well pate coated with BiOLOG metabolic substrates. Representative assay plate is depicted. Well with no substrate and positive control are boxed. Each metabolite is spotted as n = 3 in the metabolite array. Percentage of TCA substrate utilized by HLY-NT and HLY-shATM cells is depicted on the right, n = 3, *p < 0.05. (**C**,**D**) Quantitation of metabolic substrate other than TCA substrates utilization in DLBCL genetically inhibited for ATM (**C**) (sh-ATM) and (**D**) [CRISPR (CKO)] compared to WT-ATM control cells, n = 3. Experiments in (**C**,**D**) were repeated three times. Values are presented as mean + SE and asterisks define significant difference, p < 0.05.

To determine if mitochondrial dysfunction in DLBCL cells due to ATM deficiency results in compromised TCA function, we measured TCA activity using hyperpolarized (HP) [1-^13^C] pyruvate. We noticed that HLY sh-ATM (^−/−^) produces more lactate compared to HLY NT (^+/+^) indicating that conversion of HP-C1 labeled pyruvate is diverted to lactate when ATM signaling is inhibited in DLBCL cells. The mean value of lactate-to-pyruvate ratio was 2.18 × 10^–3^ ± 1.04 × 10^–4^ for HLY NT (^+/+^) and 4.46 × 10^–3^ ± 1.63 × 10^–4^ for HLY sh-ATM (^−/−^) (Fig. 4A). We also measured utilization of TCA cycle intermediates using metabolic array (MitoPlate S1, Biolog)^38^. Interestingly, we observed a decrease in utilization of TCA substrates (Fig. 4B) and a trend towards increased utilization of substrates such as, citric acid, aconitic acid glutamic acid, glutamine and tryptamine in DLBCL cell lines expressing shATM (Fig. 4C) and CKO-ATM (Fig. 4D) compared to wild type ATM-DLBCL cell lines. Thus, substrate utilization pattern of hyperpolarized (HP) [1-^13^C] pyruvate and other mitochondrial substrates were suggestive of compromised TCA function in ATM^−/−^ DLBCL compared to WT-ATM cells.

### Clinical relevance of SIRT3 in development of ATM^−/−^ DLBCL

**Figure 5:**
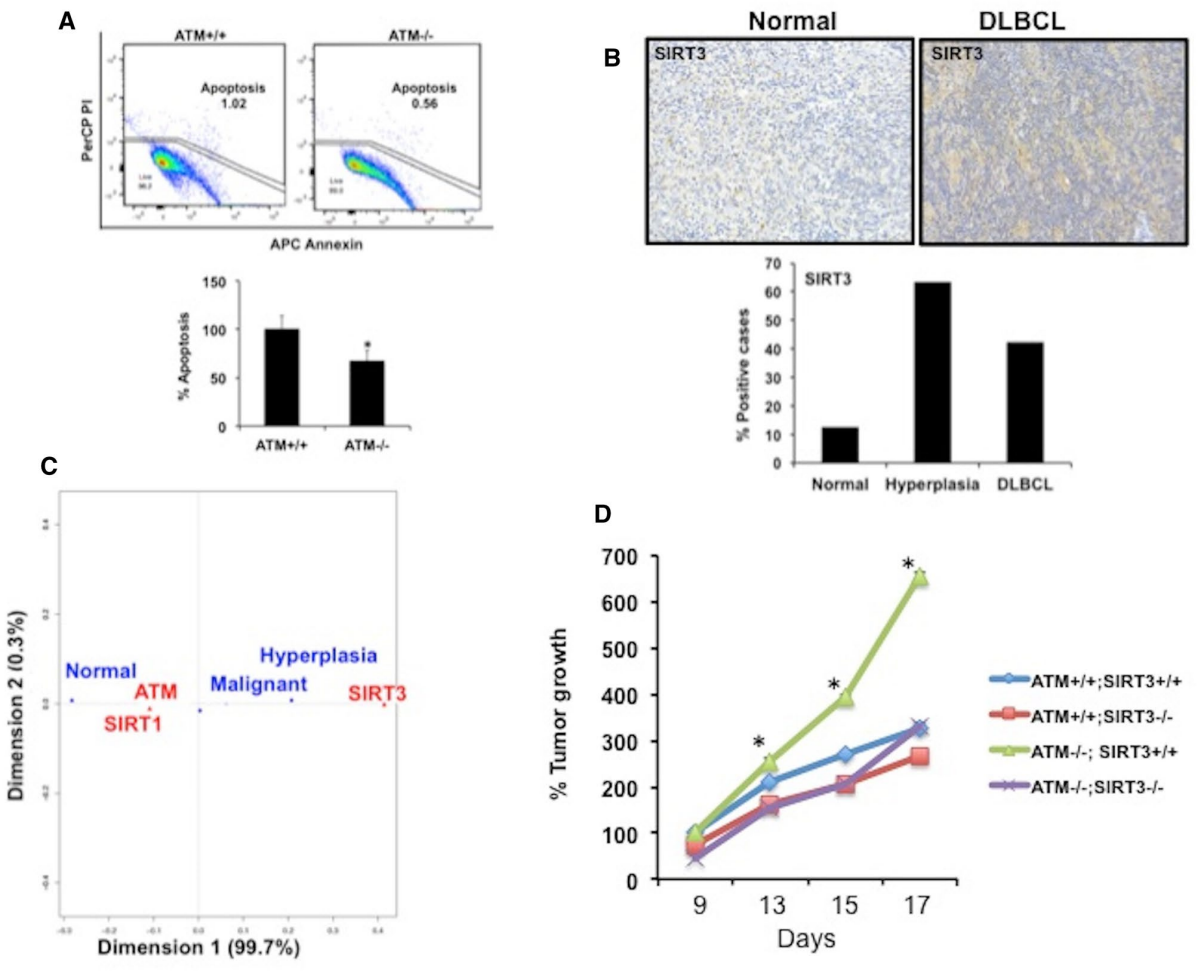
Effect of ATM deficiency and SIRT3 expression on DLBCL growth and clinical relevance in DLBCL (**A**) Representative density blot showing cell growth in DLBCL cell lines HLY (CRISPR-ATM) compared to WT-ATM control as determined by annexin V staining. Percentage apoptosis is depicted. Apoptosis observed in WT-ATM was set to 100%, p < 0.05, n = 3, + SD. Experiment was repeated three times. Representative image of dot blot is depicted in the figure. (**B**) Tumor tissue microarray (TMA) staining of DLBCL patient samples and normal controls. The percentage of tumors positive for SIRT3 staining in normal, hyperplastic and DLBCL cases is indicated. (**C**) Correspondence analysis of TMA data to establish the relationship between ATM, SIRT1, SIRT3 and DLBCL phenotype. The position of a variable from the origin on the CA graph indicates the extent of similarity of its response profile compared to the average. ATM and SIRT1 are closer to the origin, which indicates their contribution to DLBCL is small. The farther location of SIRT3 from origin implies a larger deviation from the expected contribution and its importance to DLBCL. (**D**) SIRT3 expression in DLBCL cells (HLY-WT; HLY-CKO-ATM) was inhibited using SIRT3 lentiviral particles. Growth effects of SIRT3 inhibition on tumorigenesis in NSG mice in presence and absence of ATM signaling were monitored in vivo. Tumor values were calculated from n = 10 animals per group + SE, p < 0.05.

We first explored effect of ATM expression on cell growth in vitro. Inhibition of ATM signaling in normal B-cell resulted in an increased apoptosis compared to WT-ATM cells both basally and in presence of IR stress (Supplementary Figure S5A). In contrast, CKO-ATM-DLBCL cell line HLY resisted apoptosis by 40–60% compared to that of WT-ATM cell lines (Fig. 5A). To determine if SIRT3 assists DLBCL cells to resists metabolic stress, we first determined expression of SIRT1, SIRT3 and ATM in human lymphoma samples to understand clinical relevance of these markers in DLBCL. Staining of ATM, SIRT1 and SIRT3 in lymphoma patients demonstrated a variation in their degree of expression suggesting a differential requirement of these critical markers during tumor development. We observed that percentage of DLBCL patients positive for ATM (80%) and SIRTI (87.5%) expression was similar to percentage of positive normal controls (Supplementary Fig. S5B). Strikingly, there was an increase in number of patients with hyperplasia (63%) and DLBCL (42%) that were positive for SIRT3 expression compared to positive normal controls (12.5%) (Fig. 5B). Correspondence analysis indicated that expression of SIRT3 was close to malignant group compared to ATM and SIRT1 signaling, which were closely associated to normal group (Fig. 5C). We performed additional analysis to determine prognostic importance of SIRT3 expression in survival of patients with DLBCL using Prognoscan microarray datasets^39^. Increased SIRT3 expression was associated with poor survival of DLBCL in studies depicted as GELA-221562 and GELA-221913 (Supplementary Fig. S5C). Subsequently, we directly investigated if lymphoma growth related to ATM deficiency is a result of SIRT3 stimulation. To examine this hypothesis, we inhibited endogenous SIRT3 expression using sh-SIRT3 lentivirus in CKO-ATM-HLY and WT-ATM-HLY DLBCL cell lines. DLBCL cells edited for ATM and SIRT3 expression were subsequently injected in NOD SCID gamma mouse mice (NSG) to establish xenografts. Interestingly, tumors derived from inhibition of SIRT3 signaling in presence of low ATM expression (ATM^−/−^; SIRT3^−/−^) were significantly smaller in size in contrast to ATM deficient tumors expressing endogenous SIRT3 (ATM^−/−^; SIRT3 + / +) (Fig. 5D). Overexpression of exogenous SIRT3 in ATM deficient tumors reverted growth of these tumors (ATM^−/−^; SIRT3 + / + EX) to the size similar to ATM deficient tumors expressing endogenous SIRT3 (ATM^−/−^; SIRT3 + / +) (Supplementary Fig. S5D). Tumors with WT-ATM signaling (ATM + / + ; SIRT3 + / + ; ATM + / + ; SIRT3^−/−^) grew smaller in size compared to ATM deficient tumors (Fig. 5D). Importantly, inhibition of SIRT3 has a significant impact on tumor progression. Together, this data suggest that SIRT3 is a risk-associated gene in DLBCL with low ATM expression.

## Discussion

Despite the contribution of ATM mutations in hematological malignancies, the precise knowledge of how ATM deficiency promotes DLBCL proliferation is unclear. This is the first study directly demonstrating that SIRT3 is critical metabolic target associated with lymphoma development in ATM^−/−^ background. SIRT3 stimulation was specific to ATM ^−/−^ ABC subtype of DLBCL cells, whereas, such a difference was not observed in normal ATM deficient cells compared to ATM-WT cells. Relevant to this, a recent study shows that SIRT3 is dispensable for normal germinal center B-cell and is required for DLBCL metabolic activity^22^.

ATM maintains antioxidant defense by promoting glucose flux through PPP^40^. A-T models are associated with higher levels of oxidative stress^19^. Mechanisms that regulate oxidative stress in ATM^−/−^ brain tissues are attributed to low catalase activity^41^. Although SOD2 activity is increased in cerebella of ATM^−/−^ mice^19,41^, it is unclear if increased SOD2 expression regulates ROS levels in cerebellum of mice. Oxidative stress described in ATM deficient mice models appears to be brain specific. Interestingly, ATM deficiency did not lead to ROS accumulation in lymphoma environment. Cancer cells balance ROS levels for their survival by various mechanisms including, regulation of sirtuins^42^. We suspect that SIRT3 mediated SOD2 activity is specific to cancer microenvironment in DLBCL to protect cells from oxidative stress. Our results are in agreement with a previous study in which a novel mouse lymphoma model of A-T with an N-terminal mutation in ATM showed that ATM is not necessarily associated to an oxidized phenotype^43^. Whereas, ATM null mutation in CLL results in increased ROS production, which may be likely be a cause of defect in antioxidant capacity. Expression of redox sensitive nuclear respiratory factor 2 (NRF2) was diminished in ATM^−/−^ CLL cells^13^. Therefore, it appears that the ROS phenotype of ATM^−/−^DLBCL cell is different than that observed in ATM^−/−^ CLL and may be linked to the antioxidant mechanism critical for malignant transformation in DLBCL. Our findings suggest that similar to A-T neuronal models, ATM signaling is necessary for mitochondrial integrity in DLBCL. Depressed mitochondrial respiration and stimulation of SIRT3 pathway might help to alleviate cellular load from mitochondrial dysfunction and oxidative stress for sustained tumor growth in ATM^−/−^ DLBCL.

SIRT3 is a critical mitochondrial and metabolic sensor required to maintain cellular homeostasis^44^. Metabolism of tumor cells is reprogrammed for maximum carbon consumption for their survival and proliferation^45,46^. Earlier studies suggest that ATM knockdown drives dNTP biosynthesis in IMR90 human fibroblasts^47^. Metabolic analysis of ATM^−/−^ worms and mice conducted showed differential accumulation of TCA metabolites in presence of ATM deficiency^16^. However, metabolic consequences of ATM deficiency that promotes DLBCL are not known. We observed that dependence of DLBCL on TCA substrates (pyruvic acid, alphaketoglutaric acid, succinic acid, fumaric acid and malic acid) was decreased in ATM^−/−^ DLBCL cells compared to non-target cells. Consistent with this data, shift towards lactate production was observed in sh-ATM cells compared to WT-ATM using hyperpolarized [1-^13^C] pyruvate. Similar to our observations pharmacologic inhibition of ATM with KU-55933 resulted in increased glucose derived lactate production while reduced mitochondrial respiration and accumulation of TCA cycle substrates occurred in MCF-7 cancer cells^48^. It is clear from our data that in comparison to normal physiological settings, ATM^−/−^ lymphoma cells are dependent on substrates such as citrate, aconitic acid, glutamic acid, glutamine and tryptophan for meeting metabolic requirements of proliferating tumor. In renal cancer cells where pyruvate dependent mitochondrial usage is compromised, SIRT3 supports tumor growth by promoting glutamine derived mitochondrial respiration^49^. A recent study reported that SIRT3 influence DLBCL survival by promoting glutamine addiction^22^.

GABA contents are reduced by 70% in cerebellar cortex of A-T patients, which impacts neuronal function^50^. Interestingly, our data demonstrate that GABA substrate utilization is increased in ATM^−/−^ DLBCL cells compared to wild type ATM cells. In addition to GABA utilization, RNA sequencing identified glutamate receptor and glutamine signaling as top regulated pathways in ATM inhibited dataset compared to NT-HLY cells, confirming a critical role of glutamine metabolism in development of DLBCL. In the light of our results we propose that glutamate receptor/glutamine synthesis pathway enables lymphoma cells to resist oxidative stress for sustained survival. Alphaketoglutarate (AKG) is a key substrate of TCA cycle. It also functions as antioxidant and is involved in maintaining ROS homeostasis. Although, we observed less utilization of AKG substrate in ATM^−/−^ DLBCL cells compared to NT- cells, we cannot definitively exclude the possibility of AKG being generated from glutamine, which can lead to citrate production via reductive carboxylation. AceCS2 is involved in conversion of acetate to acetyl Co.A, which can then be utilized by citric acid cycle. Since we did not observe change in AceCS2 expression in absence of ATM, we suspect ATM^−/−^ DLBCL cells may be dependent on citrate generated outside TCA cycle, for example from glutamine. Our metabolic data unravels novel metabolic alterations in ATM^−/−^ DLBCL cells and suggests that ATM^−/−^ DLBCL cells preferentially metabolize alternative energy sources other than glucose for their survival. Future studies using ^13^C-tracer glutamine will refine our understanding of how glutamine derived carbon flux impacts growth of ATM deficient DLBCL cells. To point, dependence on glutamine pathway is increased in cells with defective mitochondria to meet metabolic demand for survival^51^. Our data illustrates the clinical significance of targeting addiction to SIRT3 influenced metabolic pathways in order to impede lymphoma growth in presence of low ATM expression.

ATM mutated patients and cellular models are sensitive to IR under normal physiological stress^52,53^. Whereas, our data support a paradigm in which ATM^−/−^ DLBCL cells are resistant to apoptosis under metabolic stress. Currently, there are a number of drugs that have selective activity in ATM^−/−^ deficient cancers including inhibitors of PARP, ATR or checkpoint signaling, and nucleoside analogues such as sapacitabine^54^. However, data suggests that B-cell malignancies in A-T patients are refractory to frontline chemotherapeutic drugs that act by inducing DNA damage. Although NAD^+^ supplementation in A-T models is helpful in overcoming A-T neuropathology by improving NAD^+^/SIRT1 axis^16,55^, we suspect NAD^+^ will fuel tumor proliferation in A-T lymphoma cases associated with increased SIRT3 expression. It was recently shown that SIRT3 promotes therapeutic resistance in acute myeloid leukemia by reprograming mitochondrial metabolism towards OxPhos and by downregulating ROS generation^56^. Silencing of SIRT3 was shown to impair mitochondrial biogenesis in colon cancer cells^57^. Targeting SIRT3 dependent metabolic control of tumor cells in ATM deficient lymphoma patients will have clinical significance and likely lead to alternative novel therapeutic strategies in these patients. Indeed, our data from a preclinical tumor model demonstrated that inhibition of SIRT3 signaling lead to suppression of ATM^−/−^ xenograft tumor growth. Further, using exogenous SIRT3 expression plasmid we were able to revert growth of ATM^−/−^SIRT3^−/−^ tumors. Prognoscan database searches predicted that SIRT3 is associated with poor outcome in DLBCL cases. Regulation of SIRT4 signaling in response to genotoxic stress has been reported earlier^58^. In addition, SIRT5 suppresses DNA damage response in a ROS dependent manner via regulation of Nrf2 pathway^59^. Therefore, we also investigated if ATM deficiency impacts other two-mitochondrial sirtuins, SIRT4 and SIRT5, in DLBCL. It was shown in mice pancreatic β-cells that SIRT4 inhibits GDH activity by catalyzing the transfer of an ADP-ribosyl moiety from NAD^+^ to glutamate dehydrogenase (GDH)^60^. In contrast, we observed that GDH activity was enhanced in DLBCL cell lines in ATM deficient background. Further, pathway analysis of RNA sequence data did not show overall significant changes in SIRT4 and SIRT5 gene targets between the ATM-wildtype and ATM deficient DLBCL groups (Supplementary Fig. S6A). Real time PCR validation of critical SIRT4 target, pyruvate dehydrogenase E1 subunit alpha 1 (PDH1A) also did not show any change in expression in ATM-wild type and ATM deficient DLBCL groups (Supplementary Fig. S6B). In fact, it was recently shown that SIRT5 did not have significant impact proliferation of DLBCL compared to robust effects of SIRT3 depletion on DLBCL proliferation^22^. Together, our findings suggest that SIRT3 is mainly associated with propagation of ATM ^−/−^ DLBCL.

**Figure 6:**
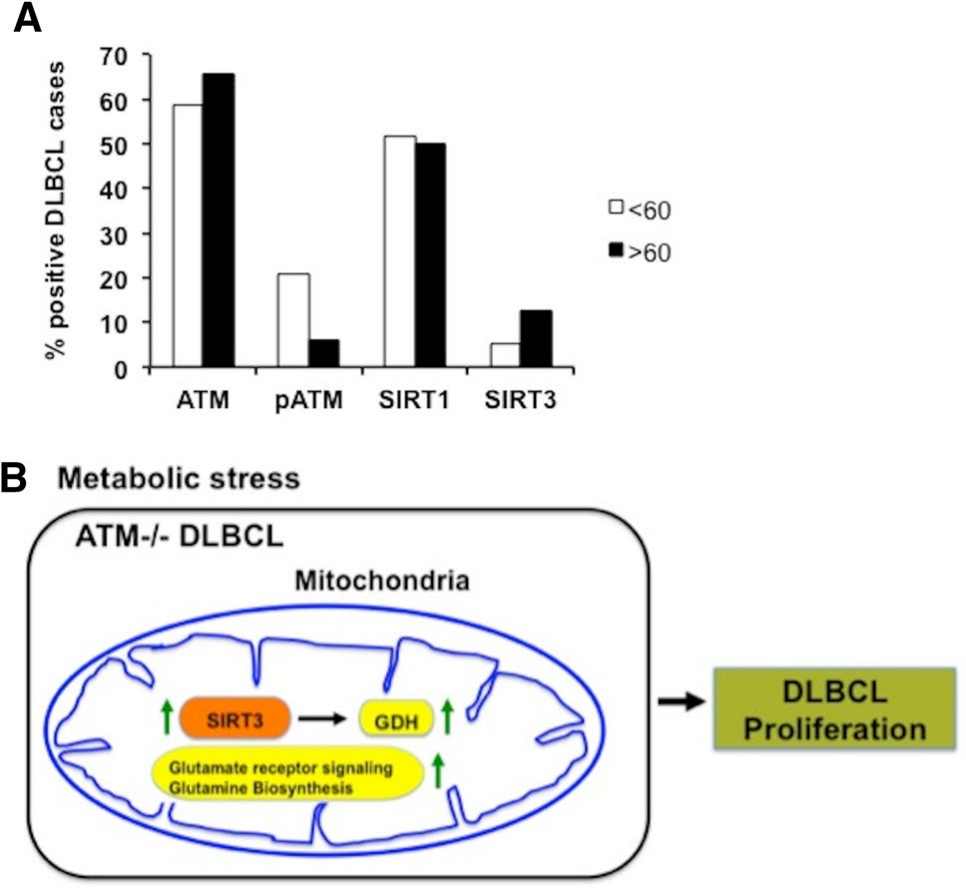
Hypothesis of how inverse relationship between ATM and SIRT3 promotes DLBCL. (**A**) Staining of DLBCL patient samples and normal controls cases spotted on TMA for ATM, pATM, SIRT1 and SIRT3. The percentage of tumors positive for staining of respective markers in normal and DLBCL cases was scored and the data was sorted by age into respective groups < 60 and > 60. (**B**) Schematic of hypothesis depicting a role of SIRT3 in ATM deficient background.

Levine and colleagues reported that function of ATM declines with age^61^. Age above 60 years is a significant adverse factor for survival of patients with non-Hodgkin’s lymphoma, including DLBCL patients^62,63^. To determine clinical relevance of induced SIRT3 expression in ATM null malignancy, we determined if there is an inverse relationship between active ATM and SIRT3 expression in primary tumors. For this purpose, we utilized tissue array expression data from primary lymphoma samples. Interestingly, only 6% of individuals were positive for active ATM (pATM) in the age group > 60 compared to 20% of individuals in the age group < 60 years. However, there was 2.5 fold increase in the number patients positive for SIRT3 expression with > 60 years of age compared to patients < 60 years of age group (Fig. 6A), revealing an inverse relationship between ATM and SIRT3 levels.

In summary, we show that enhanced SIRT3 expression promotes lymphoma growth in an ATM deficient background. As depicted in Fig. 6B, SIRT3 in mitochondria will provide an advantage to ATM deficient cancer cells by stimulating glutamate and glutamine utilization thereby promoting tumor proliferation. Therefore, considering pharmacological targeting of SIRT3 will be promising for treatment of DLBCL patients with ATM null phenotype and A-T patients with B-cell malignancies. Of relevance, inhibitors of histone deacetylase prevent ATM biding and impacts sensitivity to genotoxic stress by inducing apoptosis^64^. Recently, Melnick and colleagues developed a clinical-grade SIRT3-selective inhibitor, YC8-02, for specific targeting of mitochondrial SIRT3 in DLBCL because SIRT3 was found to be dispensable for normal cells^22^. Selective inhibition of SIRT3 expression using YC8-02 specifically killed DLBCL cells both in vitro and in vivo. However, in the light of previous studies, which suggest that SIRT3 also functions as a tumor suppressor^44^, the use of SIRT3 inhibitors needs to be employed cautiously. Downstream targeting of SIRT3 influenced metabolic pathways will be more efficient in patients with DLBCL where ATM expression is low. In the aggregate these data provide a foundation for novel therapeutic strategies that can be targeted independently of the DDR pathway in ATM-deficient subset of DLBCL, A-T patients, and elderly patients with DLBCL where ATM expression is low.

## Methods

### Cell culture and reagents

DLBCL cell lines were obtained from American type culture collection (ATCC). DLBCL cells were cultured in RPM1 medium (Gibco, MD, USA) and media was supplemented with 100 units/ml penicillin, 0.1 mg/ml streptomycin and 10% FBS. HLY-1 cells were obtained from Dr. Ari Melnick (Weill Cornell, NY). B-cell lymphocyte cell line and ATM^−/−^ fibroblast cells were purchased from Coriell Cell Repositories. Cells were subjected to IR stress with dose of 4 Gy using a Pantak Seifert X-ray machine. Cells were irradiated at 250 kV, 13 mA as described previously^25^. Cells were irradiated at room temperature and cultured for 2 h to overnight at 37 °C. Live and dead cells were distinguished using APC Annexin V (Invitrogen, CA, USA) and ROS levels were measured using Cell ROX (C10443) (Molecular Probes). Flow cytometry was performed using FACS Canto-II.

### RNA and protein expression

Total cytoplasmic RNA was isolated from DLBCL cell lines and RT-PCR was performed as previously described^14^. Western blotting was performed as per standard methods. Western blot data presented in all figures is a representative of independent experiments that were initiated generally from three independent cell cultures. Protein expression was quantitated using Image J software. For accurate quantitative analysis, within each experiment, gel band on each blot was measured in triplicate using Image J. This minimizes error and variability while calculating relative abundance for respective protein expression from western blot analysis. Average value of three independent measures is provided as percentage expression of target protein. Protein expression was normalized using appropriate controls in respective experiments. Primary antibodies were used at a dilution of 1:1000 and secondary antibodies 1:10,000 dilution. Following antibodies were used in the current study: ATM (D2E2, Cell Signaling Technology), SIRT1 (SC-15404, Santa Cruz), SIRT3 (SC-365175, Santa Cruz), FOXO3A (75D8) (Cell Signaling Technology 2497), GAPDH (6C5) (abcam, ab8245), Tom20 (612278 BD Transduction Laboratories), VDAC (D73012, Cell Signaling Technology), Cytochrome C (EPR1327) (abcam, ab133504), H3 (Cell Signaling Technology, 9715), Acetylated lysine (9441, Cell Signaling Technology), MRPL10 (abcam, ab229097), SOD2 (E-10) SC-137254, Santa Cruz), SOD2 (acetyl K68) (EPVANR2, abcam), GDH (D9F7P, Cell signaling Technology), OPA1 (612606, BD Transduction Laboratories), NDUFA9 (abcam, ab14713), DRP1 (BD Transduction Laboratories, 611113), Acetyl CoA synthetase (abcam, EPR8500). Secondary antibodies were purchased from spectra for immunofluorescence studies*.*

### Cellular fractionation

Mitochondrial fractionation was performed using mitochondrial isolation kit for culture cells (Sigma-Aldrich, Saint Louis, Missouri, USA). Briefly, DLBCL cells were subjected to Dounce homogenization and mitochondrial pellets were isolated lysed as per manufacturer’s instructions. Nuclear isolation was performed using CellLytic NuCLEAR Extraction kit as per the recommended instructions (Sigma-Aldrich). Mitochondrial and nuclear lysates were subsequently used for immunoblotting.

### RNAseq analysis

RNAseq analysis (BioProject ID: PRJNA605211) was carried out by the Informatics Resource Center, Institute for Genome Sciences, UMBSOM using Paired-end illumina libraries. Polysomal RNA was isolated from DLBCL cell lines expressing wild type ATM and shATM as previously described^14^. RNA quality control, library preparation, sequencing and analysis were performed using standard operating procedures of the core. Following library preparation, libraries were multiplexed in groups of 4 per flow cell lane using a 150 bp paired-end run. Each flow cell lane on the illumina HiSeq4000 platform yields > 312 million read pairs which represents more than 50 million read pairs per sample and provides a considerable depth of sequencing coverage. Paired-end illumina libraries are mapped to the human reference, Ensemble release GRCh38.96, using HiSat2 v2.0.4, using parameters previously described^65–69^. Read counts for each annotated gene are calculated using HT-Seq. The DESeq Bioconductor package (v1.5.24) is used to estimate dispersion, normalize read counts by library size to generate the counts per million for each gene, and determine differentially expressed genes between disease and control samples. Differentially expressed transcripts with an FDR ≤ 0.05 and log_2_ fold change ≥ 1 are used for downstream analyses. Normalized read counts are used to compute the correlation between replicates for the same condition and compute the principal component analysis for all samples. The list of differentially expressed genes is used to compute the enrichment of biological pathways using Ingenuity Pathway Analysis (IPA).

### CRISPR knockdown of ATM

Genetic inhibition of ATM was done using human shRNA lentiviral particles (TL320267V) from OriGene Technologies (Rockville, MD, USA). Genetic knockdown of SIRT3 and overexpression of SIRT3 in DLBCL cell lines was carried out using LPP-HSH006079-LVRU6MP-100 and LPP-UI445-Lv216-100 lentiviral particles respectively from OriGene Technologies (Rockville, MD, USA). ATM CRISPR knockout in DLBCL cell line HLY was performed as previously described^70^ using ATM exon 7 targeting sgRNA AGTTGACAGCCAAAGTCTTG. Generated ATM-KO single cell clones were confirmed by NGS.

### Immunoprecipitation (IP)

Lymphocytes were harvested in lysis buffer containing 150 mM KOAc, 2.5 mM Mg(OAc)_2_, 20 mM K-N-2-hydroxyethylpiperazine-N′-2-ethanesulfonic acid, pH7.5, dithiothreitol, phenylmethlysulfonly fluoride, RNasin, and protease inhibitors. For IP reactions 500ug for mitochondrial lysates was used. Cells were harvested with buffer containing 10 mM nicotinamide (NAM) (Sigma-Aldrich, USA) and Sodium butyrate (Sigma-Aldrich, USA) to probe for acetylated proteins. Other reagents used for detection of IP proteins included: Clean blot IP detection kit (HRP) ThermoFischer Scientific (USA), Rabbit True Blot (18-8816-31, ROCKLAND antibodies and assays USA), and Anti-rabbit IgG magnetic beads (00-1800-20, ROCKLAND antibodies and assays USA).

### Glutamate dehydrogenase (GDH) activity

GDH *activity* was measured in DLBCL cells using GDH kit (abcam, ab102527) (MA, USA) as per manufacturer’s instructions. GDH activity was determined colorimetrically (λ = 450 nm).

### Mitochondrial structure and function

Mitochondrial structure was determined using Electron Microscopy. Cells were collected and fixed in a solution of 2% paraformaldehyde, 2.5% glutaraldehyde, in 0.1 M PIPES buffer (pH 7) at room temperature for one hour. After washing, cells were quenched with 50 mM glycine in 0.1 M PIPES buffer (pH 7) for 15 min, pelleted and enrobed in 2.5% Low melting point agarose. Agarose blocks containing cells were trimmed into 1mm3 blocks and post-fixed with 1% osmium tetroxide and 1.5% potassium ferrocyanide in 0.1 M PIPES buffer for 1 h at 4 °C, washed, stained with 1% uranyl acetate in water and dehydrated using increasing concentration of ethanol from 30%; 50%; 70%; 90% and 100% for 10 min at each step. Specimen were then incubated with two changes of 100% acetone and infiltrated, in increasing concentration of Araldite-Epoxy resin (Araldite, EMbed 812; Electron Microscopy Sciences, PA), and embedded in pure resin at 60 °C for 24–48 h. Ultrathin sections at ~ 70 nm thickness were cut on Leica UC6 ultramicrotome (Leica Microsystems, Inc., Bannockburn, IL), and examined in a FEI Tecnai T12 electron microscope operated at 80 kV. Digital images were acquired by using a AMT bottom mount CCD camera and AMT600 software. Mitochondrial images were obtained from different slide sections of multiple cells. Mitochondrial length and width were measured using Image J. Mitochondrial respiration was measured as described previously^14^. Assessment of mitochondrial mass in ATM^+/+^ and ATM^−/−^ DLBCL cell line HLY was measured using MitoTracker Green by flow cytometry using method published earlier^14,71^. Cells were treated with Mito Tracker Green for 30 min. Subsequently, fluorescence of mitotracker green was determined by flow cytometry. Mitochondrial oxygen consumption rate was measured in WT-ATM and ATM inhibited DLBCL cell lines as previously described^14^. Briefly, 5 × 10^5^ cells were cultured in 200μl of seahorse media with 10 mM glucose, 2 mM glutamine, 1 mM pyruvate in XF24 V7 plates under regular culture conditions and oxygen consumption measurements were performed using an XF24 Extracellular Flux Analyzer and Wave software (Agilent Technologies, Santa Clara, CA, USA). Prior to cell plating, XF24 V7 plates were coated with the cell adhesive Cell-Tak (BD Biosciences, Bedford, MA, USA) as per manufacturer’s instructions. 25 µM of 2,4-dinitrophenol (DNP) was added per injection to stimulate maximal respiration. The Complex III inhibitor Antimycin A (1 µM) was added last and any remaining respiration subsequent to antimycin A addition was considered non-mitochondrial respiration.

### Hyperpolarized [1-13C] pyruvate

For metabolic analysis, we used [1-^13^C] pyruvic acid (Sigma-Aldrich, Miamisburg, OH) hyperpolarized to 40–50% via dynamic nuclear polarization as described in^72^. Subsequently ^13^C-MRS measurements of the HP pyruvate solution were performed as described in^73^. Cell pellets were prepared for labeling of (HP) [1-^13^C] hyperpolarized pyruvate. Briefly, DLBCL cells were seeded in RPM1 and cultured under regular culture conditions. We used 100 × 10^6^ cells per hyperpolarized experiment. Cells were pelleted and suspended in 2 ml of culture media. This 2 ml of cell suspension was then placed in a 50 ml falcon tube and 2 ml of hyperpolarized pyruvate solution was added. Subsequently imaging was performed to detect incorporation of pyruvate-labeled carbon in the TCA as previously described^72,73^.

### BiOLOG screening

TCA function was identified in ATM^−/−^ DLBCL cells using MitoPlate S-1 assay (BiOLOG, CA, USA) as per protocols described in^38^, which were modified to use in lymphoma cells. BiOLOG assay is a colorimetric-based method, which utilizes redox dye and provides a high-resolution approach to measure mitochondrial function. Briefly, cells were permeabilized with permeabilizing buffer, followed by, subsequent addition of dye mix from (BiOLOG, CA, USA). Formation of air bubbles upon dye mix addition was carefully avoided. We used 50,000 cells/well for BiOLOG MitoPlate assays. Beginning after a one-hour incubation period, color measurement was captured every 15 min for two hours using a plate reader (Synergy H1M) (BioTek, Winooski, VT, USA) that allowed kinetic reading at 590. Data was plotted as percentage utilization of substrate in ATM^−/−^ cells compared with cells expressing wild-type ATM.

### Immunohistochemistry (IHC)

Antibodies used for IHC included SIRT1 (E104, NB110-57573, NOVUS BIOLOGICALS), ATM (2C1), GeneTex, SIRT3 (SC-365175). Tissue microarrays (TMAS) (LY800a and LY800b) were purchased from Biomax (MD, USA). Tissue samples from DLBCL, follicular lymphoma, reactive hyperplasia and normal controls are spotted on these arrays. Each array included 40 DLBCL samples, 5 follicular samples, 15 reactive hyperplasia samples and 20 normal samples. LY800a and LY800b TMAS were stained with ATM, SIRT1 and SIRT3 antibodies by Mass histology (MA, USA).

### Xenograft studies

Animals were subcutaneously injected in flank with DLBCL cell lines genetically edited for ATM and SIRT3 function. These cell lines consisted of following groups: ATM^+/+^/; ATM CKO; ATM^+/+^/SIRT3^+/+^; ATM^+/+^, SIRT3^−/−^; ATM CKO/SIRT3^+/+^; ATM CKO SIRT3^−/−^ and ATM CKO/(Ex)-SIRT3^+/+^. 1 × 10^6^ cells from respective groups were suspended in matrigel and the mixture was subcutaneously injected into the left and right dorsal flanks of NSG male mice. Progression of xenografts tumors was monitored three times a week until tumor volume reached 2 cm^3^.

### Statistical analysis

Mitochondrial length and width was visualized using Beeswarm Box plot analysis. All statistical analyses were performed using Student t-test and were expressed as + (SD) standard deviation of mean. Correspondence analysis was performed using CA package of the statistical language R. SIRT3 expression in DLBCL studies were analyzed using Prognoscan^39^.

### Data sharing

For original data please contact kbhalla@som.umaryland.edu. RNA sequencing data are available at GEO number GSE145359.

Xenograft studies in NSG mice (Jackson Labs, USA) were performed by the translational core facility at the University of Maryland as per the regulations of Institutional Animal Care and Use Committee of University of Maryland, Baltimore, MD, USA. The animal protocol (0819006) for translational core, University of Maryland Greenebaum Cancer Center was approved by the Institutional Animal Care and Use Committee of the University of Maryland. All other experimental protocols used in the current study were performed as per the regulations of Environmental Health and Safety guidelines of University of Maryland, Baltimore, MD, USA.

## Supplementary information

Supplementary Information.
