# Supplementary material for: SIRT3, a metabolic target linked to ataxia-telangiectasia mutated (ATM) gene deficiency in diffuse large B-cell lymphoma
Source: Sci Rep. 2020 Dec 3;10:21159. doi: 10.1038/s41598-020-78193-6 (PMC7712916; doi:10.1038/s41598-020-78193-6)

**SI information:**

**SIRT3, a metabolic target linked to Ataxia-Telangiectasia mutated (ATM) gene deficiency in Diffuse Large B-cell Lymphoma**

Kavita Bhalla^1,^*****, Sausan Jaber^2^, Kayla Reagan^1^, Arielle Hamburg^1^, Karen F. Underwood^1^, Aditya Jhajharia^3^, Maninder Singh^3^, Binny Bhandary^1^, Shambhu Bhat^1^, Nahid M. Nanaji^4^, Ruching Hisa^5^, Carrie McCracken^6^, Heather Huot Creasy^6^, Rena G. Lapidus^1^, Tami Kingsbury^7^, Dirk Mayer^3^, Brian Polster^2^, Ronald B. Gartenhaus^8^

^1^Marlene and Stewart Greenebaum Comprehensive Cancer Center, University of Maryland, Department of Medicine, Baltimore MD 21201, USA. ^2^University of Maryland, Department of Anesthesiology, Baltimore MD 21201, USA. ^3^Department of Diagnostic Radiology and Nuclear Medicine, University of Maryland, Baltimore, MD, United States. ^4^Veterans Administration Medical Center, Baltimore, MD 21201, USA. ^5^Electron Microscopy Core Imaging Facility, University of Maryland, Department of Medicine, ^6^Institute of Genome Sciences, University of Maryland. ^7^Department of Physiology, the Center for Stem Cell Biology & Regenerative Medicine, Baltimore MD 21201, USA. ^8^Hunter Holmes McGuire Veterans Administration Medical Center, Virginia Commonwealth University, School of Medicine, VA, USA.

**SI Figures and Legends:**

**
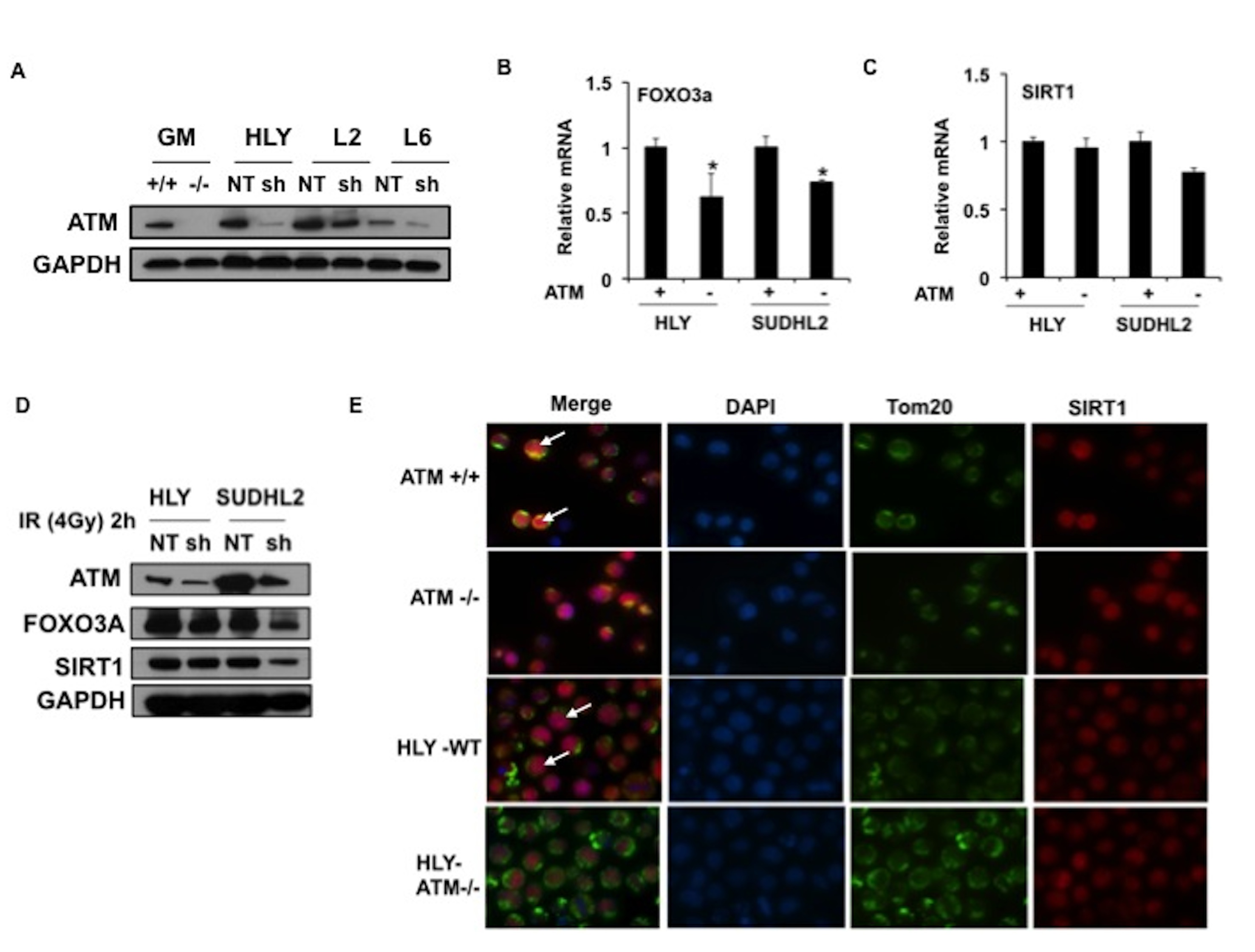
**

**Figure S1. FOXO3A and SIRTI expression in DLBCL cell lines. (A)** Expression of ATM in normal GM control cell lines and genetically inhibited DLBCL cell lines HLY, SUDHL2 (L2), SUDHL6 (L6). The gel images presented here are representative of three independent experiments. **(B)** RNA expression of FOXO3A in DLBCL cell lines HLY and SUDHL2. **(C)** RNA expression of SIRT1 in DLBCL cell lines HLY and SUDHL2. ATM (+) and ATM(-) represents DLBCL cell lines expressing non target (NT)-shRNA and ATM-shRNA lentivirus. **(D)** Western blot analysis of FOXO3A and SIRT1 expression in NT-shRNA and shRNA-ATM expressing DLBCL cell lines (HLY and SUDHL2) in presence of genotoxic stress (IR, 4Gy). The gel images presented here are representative of three independent experiments. **(E)** Immunofluorescence staining to detect nuclear SIRTI expression using SIRT1 antibody. DAPI was used to stain nucleus and Tom20 for localization of mitochondria. Top panel (ATM +/+) represent staining of normal B-cell, GM02184. ATM-/- images are from staining of ATM deficient control fibroblast cell lines (GM03332). ATM signaling was inhibited in HLY cell line using ATM kinase inhibitor (KU-55933). Parental HLY cell line was used as wild-type ATM control. SIRT1 expression (Red color) in merged images of ATM +/+ is indicated by white arrows. The images are representative of two experiments performed under similar experimental conditions.

**
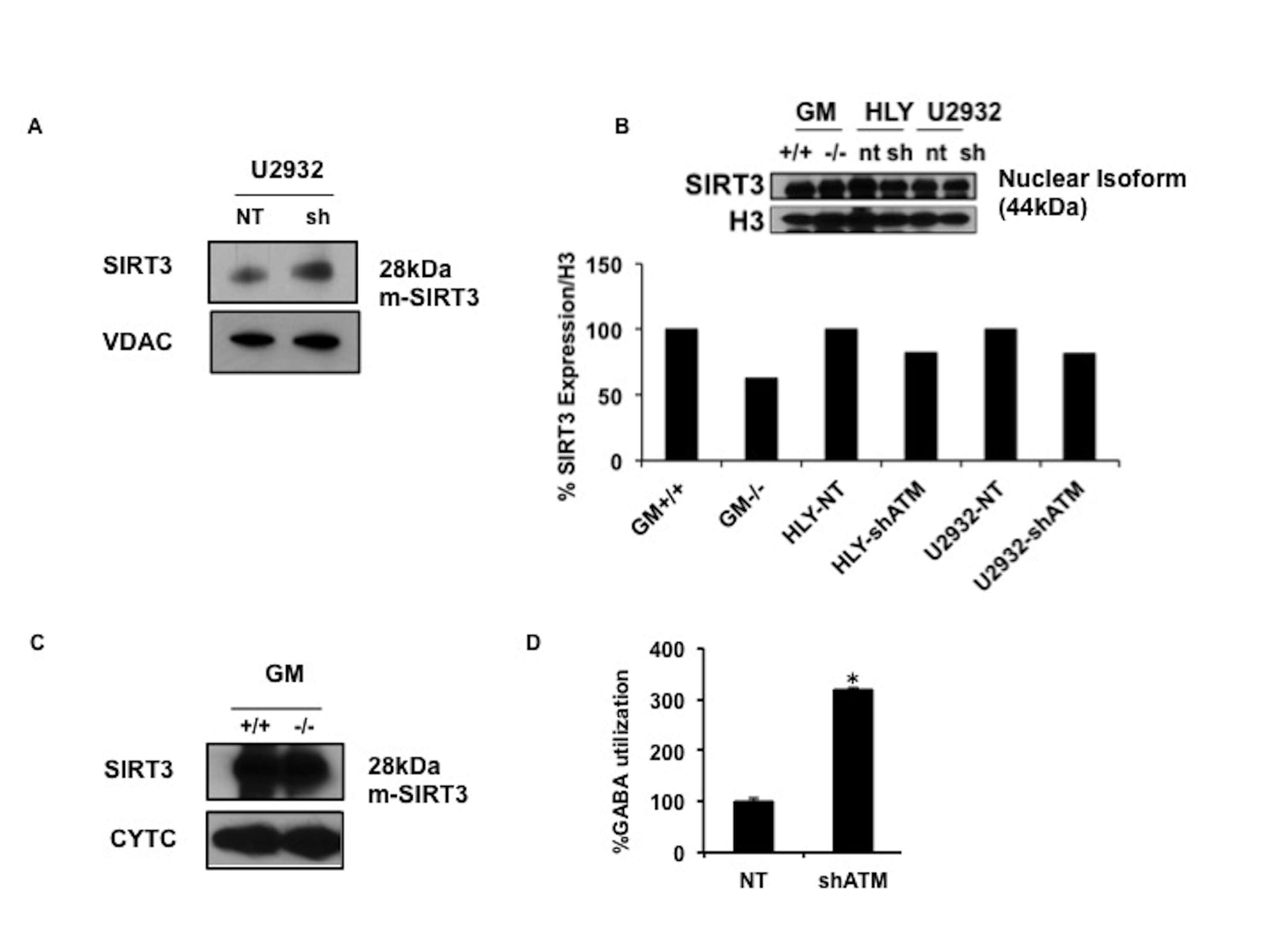
**

**Figure S2. ATM, GABA and SIRT3 expression in GM and DLBCL cell lines.** **(A)** SIRT3 expression in DLBCL cell line U2932. The data presented is a representative of three independent experiments. **(B)** Expression of nuclear SIRT3 (44kDa) in normal GM control cell lines and ATM inhibited DLBCL cell lines HLY and U2932. The data presented is a representative of two independent experiments. **(C)** SIRT3 expression in normal GM control cell lines. Size for mitochondrial isoform of SIRT3 (28kDa) is depicted. The data presented is a representative of two independent experiments. **(D)** Utilization of metabolic substrate γ-aminobutyric acid in ATM inhibited DLBCL cell lines HLY compared to non-target control, n=3, *p<0.05.

**
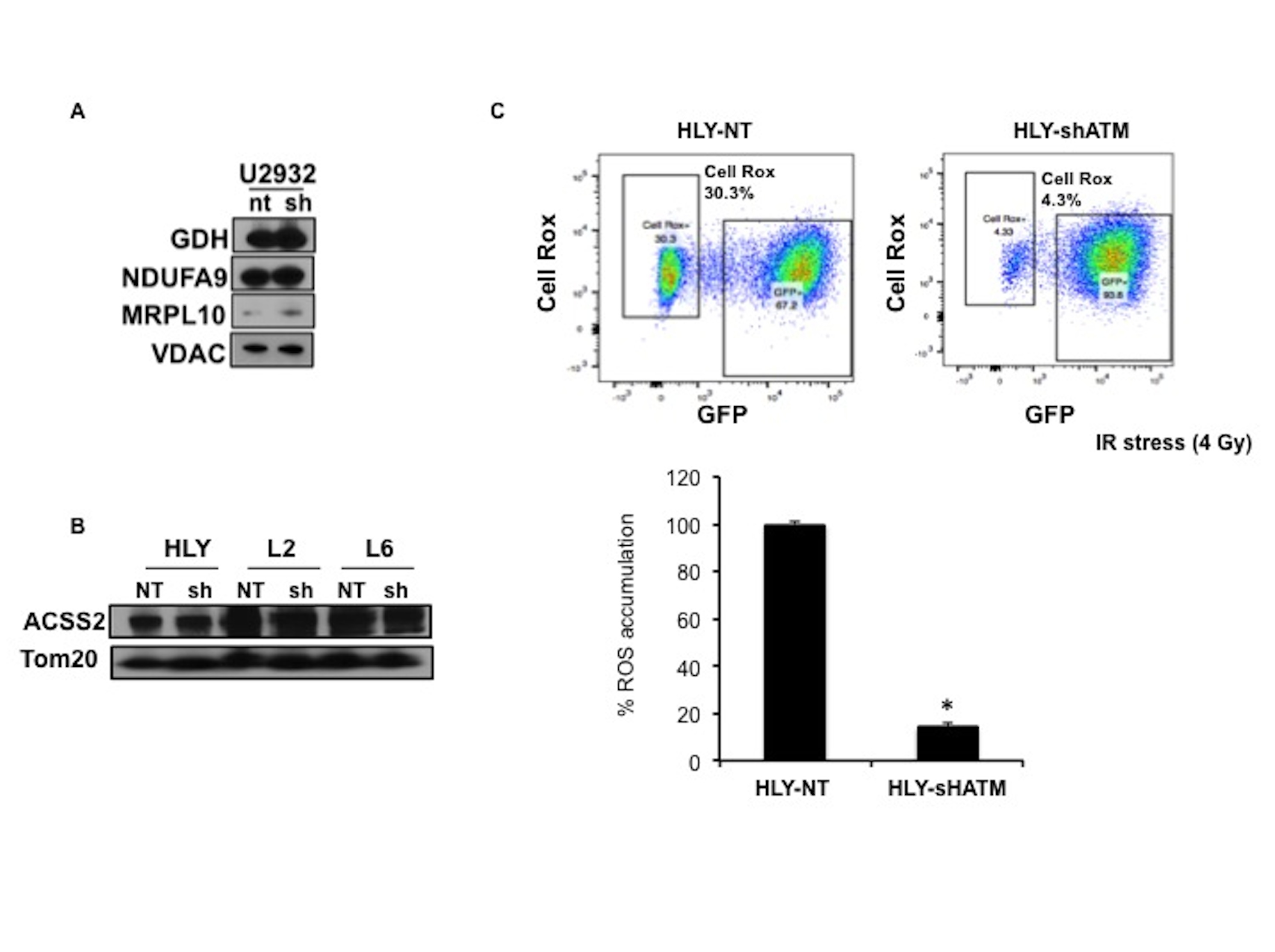
Figure S3. SIRT3 target ACSS2 and ROS. (A)** Expression of SIRT3 targets in DLBCL cell line U2932 inhibited for ATM expression compared to non-target controls. Gel images are representative blots from three independent experiments. **(B)** Expression of SIRT3 target acetyl CoA synthetase short chain family member 2 (ACSS2) in sh-ATM DLBCL cell lines HLY, SUDHL2 (L2) and SUDHL6 (L6) compared to non-target expressing controls. Gel images are representative blots from three independent experiments. **(C)** Top panels represents dot blot from FACs analysis of CellROX labeled DLBCL cells. Quantitation of ROS accumulation in DLBCL cell line HLY stably expressing NT-GFP and shATM-GFP lentivirus, n=3, *p<0.05. Experiment was repeated three times and representative images are depicted in the figure.


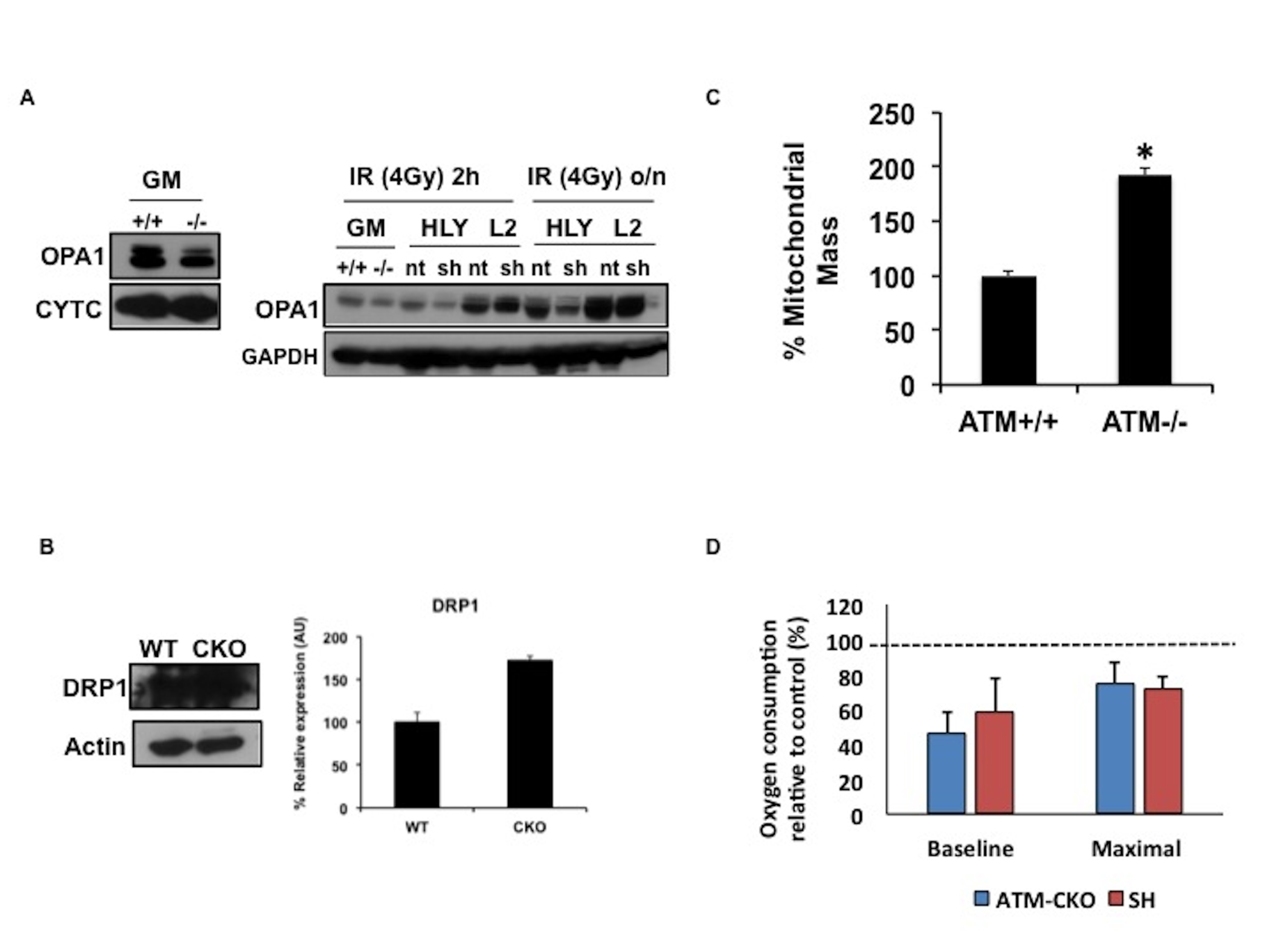


**Figure S4. Western blot analysis and OCR traces. (A)** Expression of OPA1 in normal ATM +/+ (GM02184) and ATM-/- (GM03332) GM cells**.** OPA1 expression in DLBCL cells transduced with NT-shRNA and sh-ATM lentivirus. Cells were exposed to IR stress (4Gy), subsequently incubated for 2 hours and overnight, followed by protein isolation in respective case. Western blotting was done using OPA1 antibody. GM cells were used as control in the experiment. Gel images are representative blots from two independent experiments. **(B)** DRP1 expression in genetically inhibited HLY cells compared to WT-ATM control. Gel images are representative blots from two independent experiments. **(C)** Mito Tracker green staining to measure difference in mitochondrial mass in presence of ATM deficiency compared to ATM wild type DLBCL cell line HLY. Each experiment was set as n=3, asterisks define significance difference, p<0.05. **(D)** Maximal and basal respiration rates in cell inhibited for ATM using CRISPR (CKO) and lentiviral (SH) particles. Respiration was set to 100% in cells with WT-ATM. Each experiment was set using n=5 to 6 replicates per ATM+/+ and ATM-/- cells. Graphs are represented as mean +SD of three independent experiments.

**
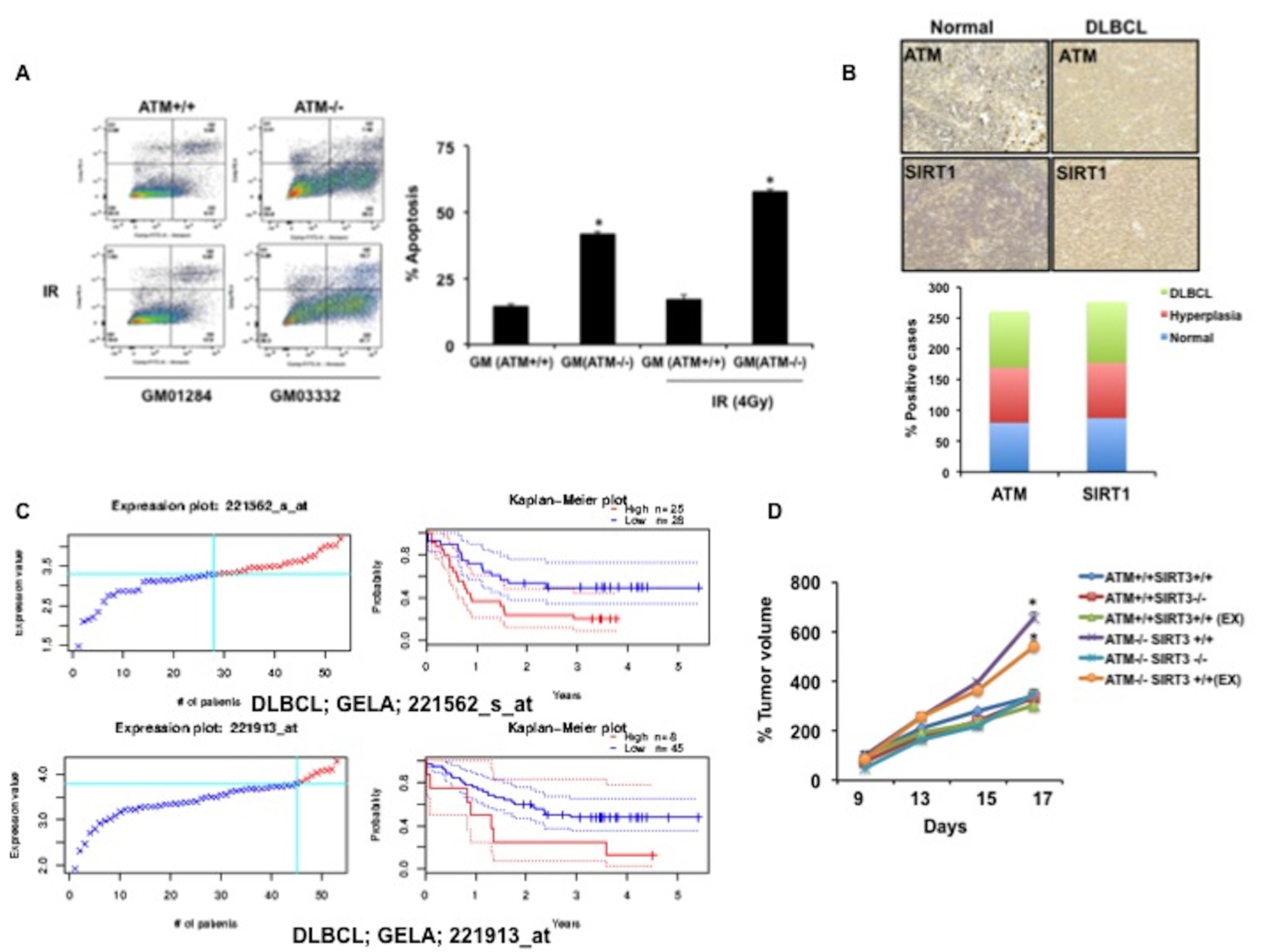
**

**Figure S5. ATM, SIRT1 and SIRT3 in DLBCL. (A)** Effect of ATM expression on cell growth in presence of IR in ATM+/+ and ATM-/- normal control (GM) cells. Cells were stained using Annexin V to measure apoptosis by flow cytometry, *p<0.05 (n=3). **(B)** Lymphoma tissue microarray screening for ATM and SIRT1. Positive percentage tumor for respective patient groups and normal controls for ATM and SIRT1 staining is depicted below. **(C)** Kaplan Meier analysis of the DLBCL dataset showing SIRT3 is linked to poor DLBCL survival. **(D)** Exogenous SIRT3 (EX) was ectopically expressed in ATM-WT and ATM-/- DLBCL cell line HLY. Growth effect of (EX) SIRT3 was compared to xenograft tumors established using genetically inhibited ATM and WT-ATM DLBCL cell lines that were inhibited for SIRT3 signaling. Tumor values were calculated from n=10 animals per group +SE, p<0.05.

**
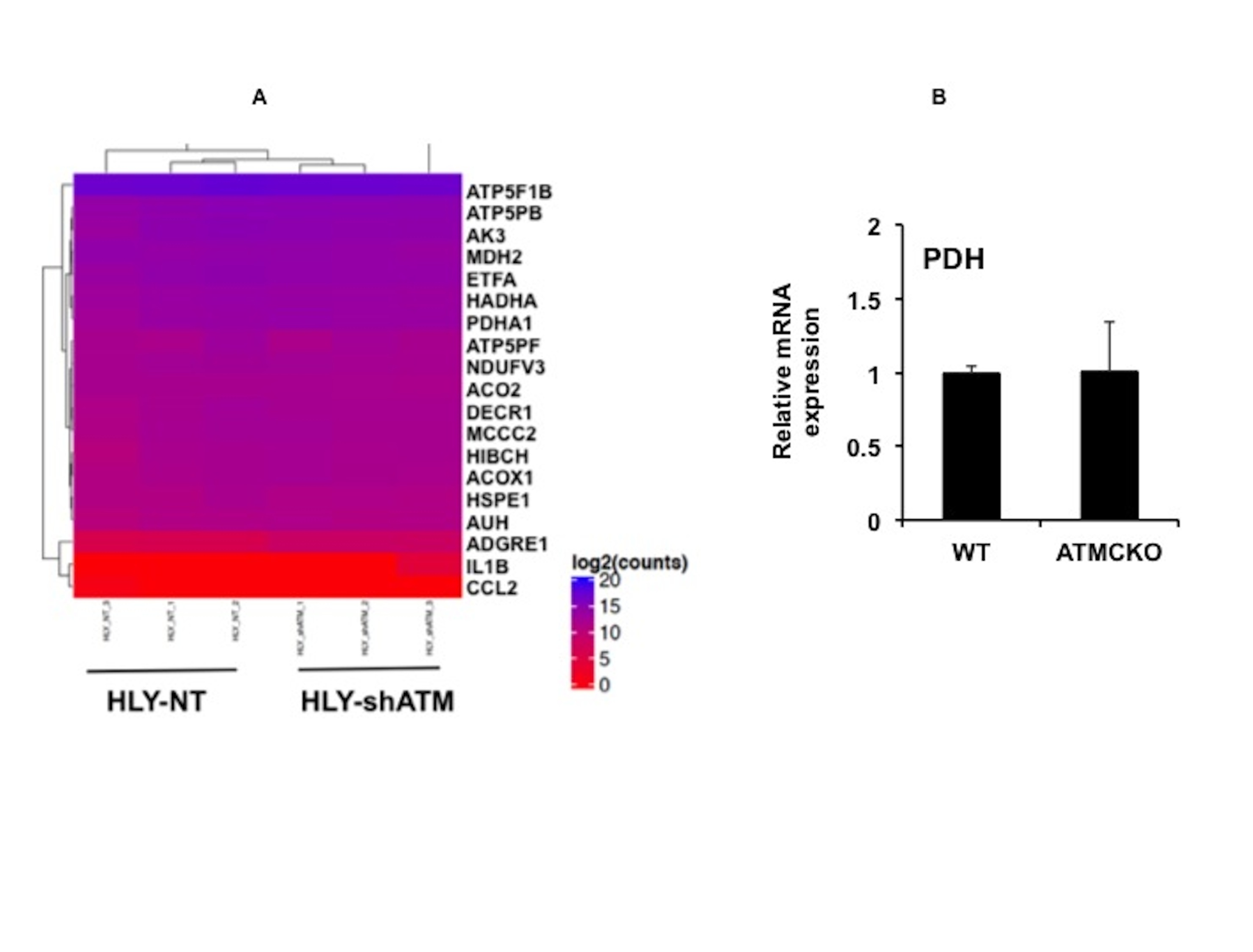
**

**Figure S6. Effect of ATM deficiency on regulation of SIRT4 and SIRT5 signaling. (A)** Gene clustering illustrated by a heatmap of SIRT4 and SIRT5 targets across samples in shATM cell line compared to its non-target control (NT) (n=3/group). Gene expression values come from log2 raw counts generated by htseq-count. Heat map was created using the R library Complex Heatmap, which is part of Bioconductor software (3.1): [http://bioconductor.org/packages/release/bioc/html/ComplexHeatmap.html](http://bioconductor.org/packages/release/bioc/html/ComplexHeatmap.html). No overall significant changes in SIRT4/5 targets were observed between the two groups ATM-WT and ATM-/- DLBCL. **(B)** Real time PCR validation of critical SIRT4 target pyruvate dehydrogenase E1 subunit alpha 1 (PDH1A) in ATM-wild type and ATM deficient DLBCL cell line HLY. RT-PCR analysis was set as n=3 for both control and ATM -/- DLBCL cells.


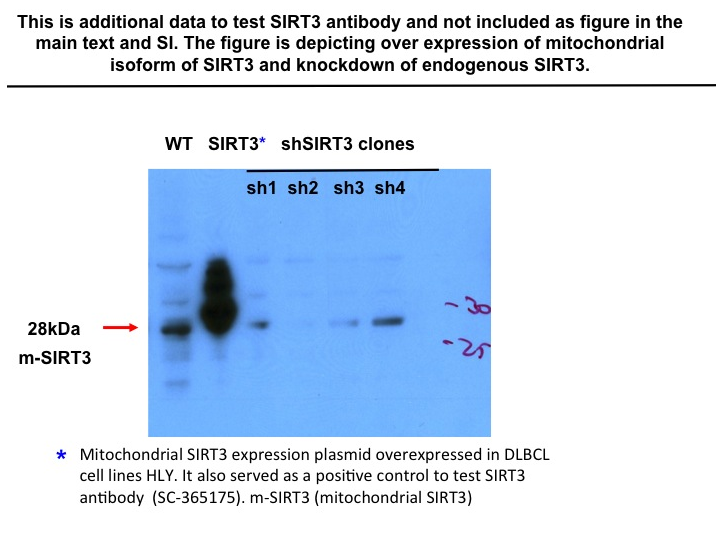


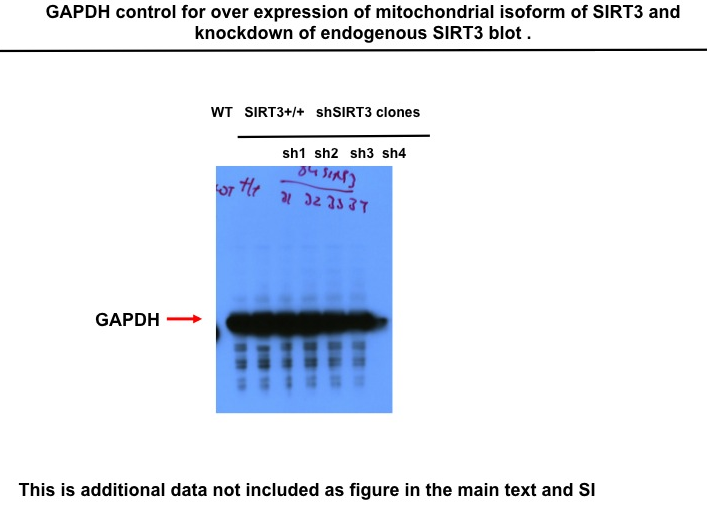

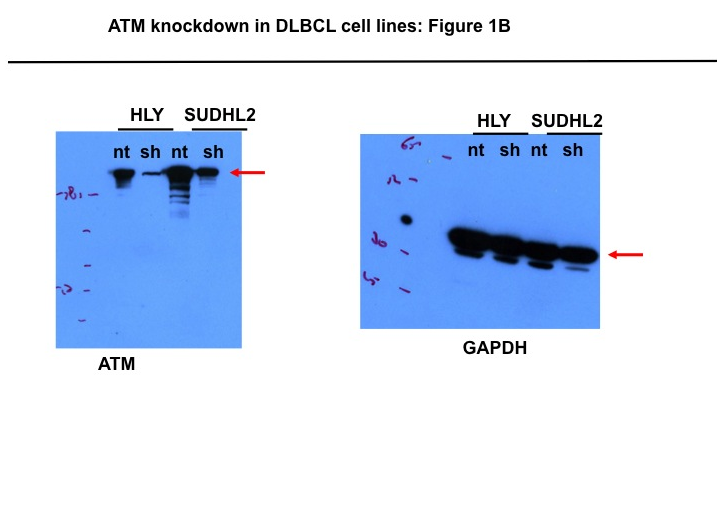

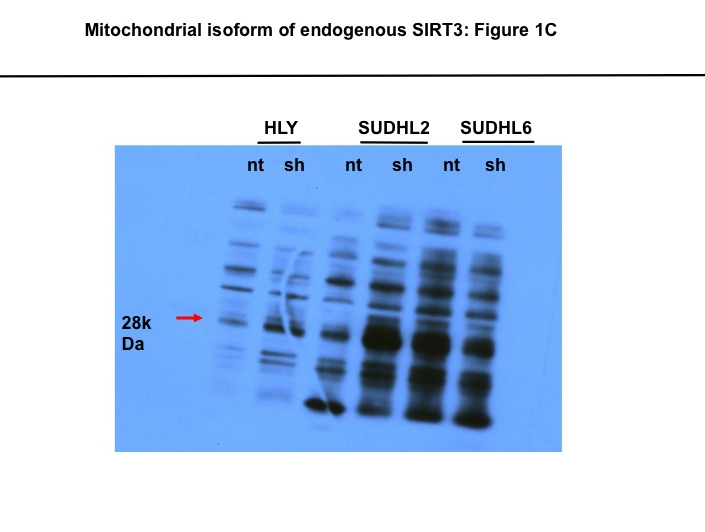

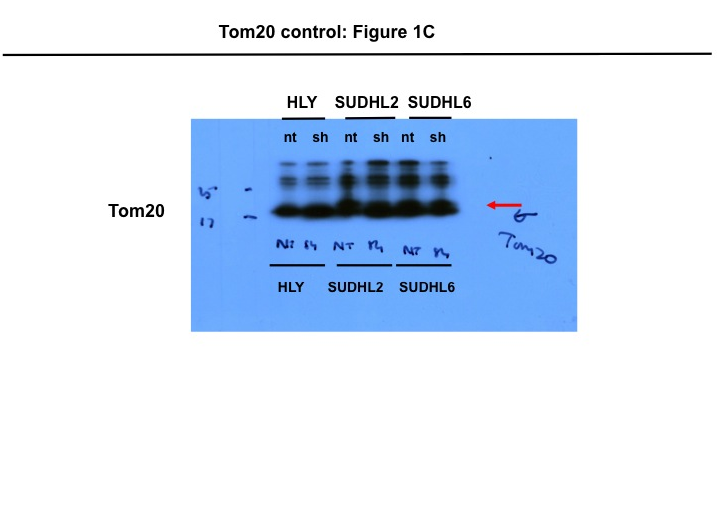

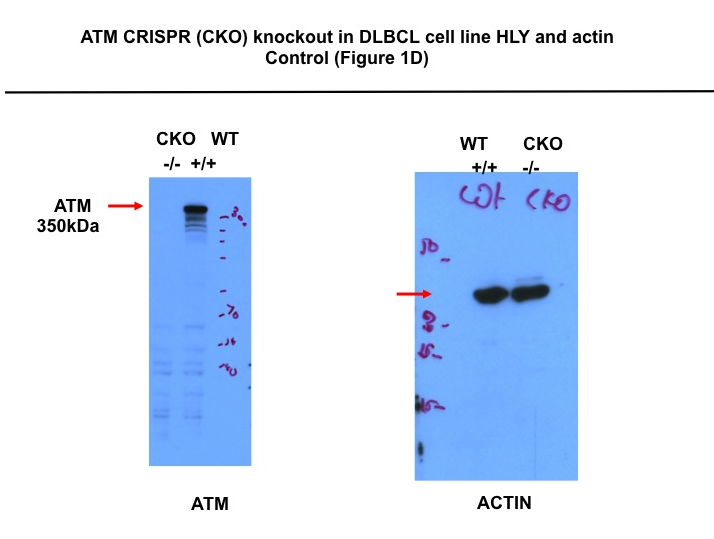

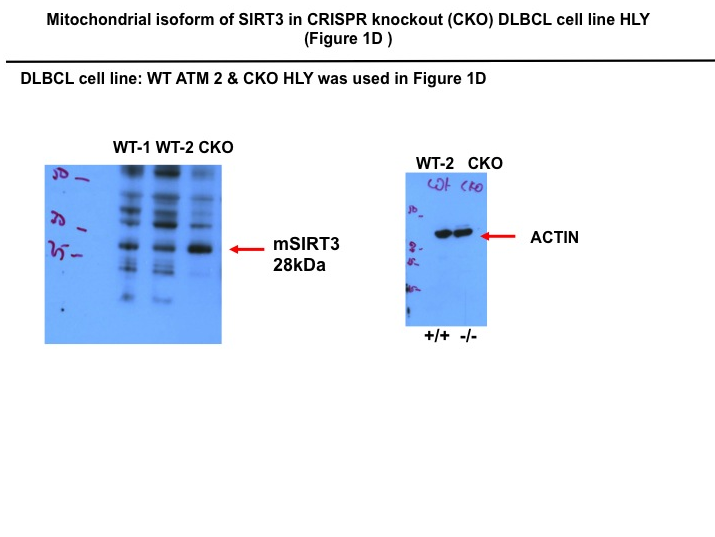

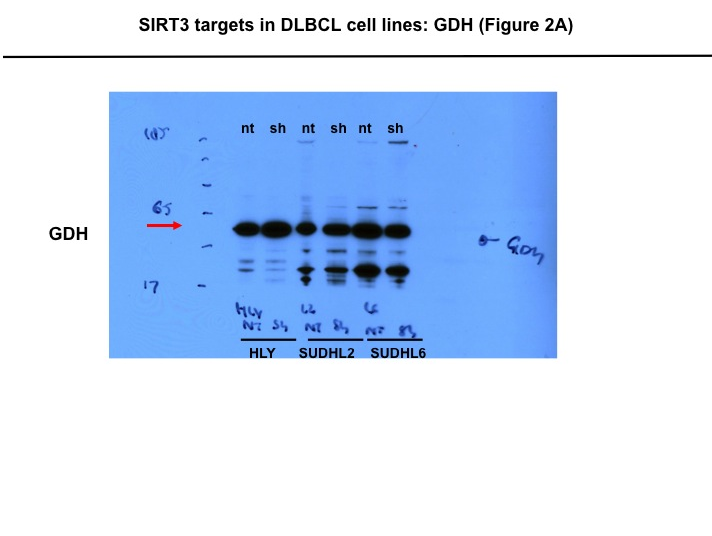

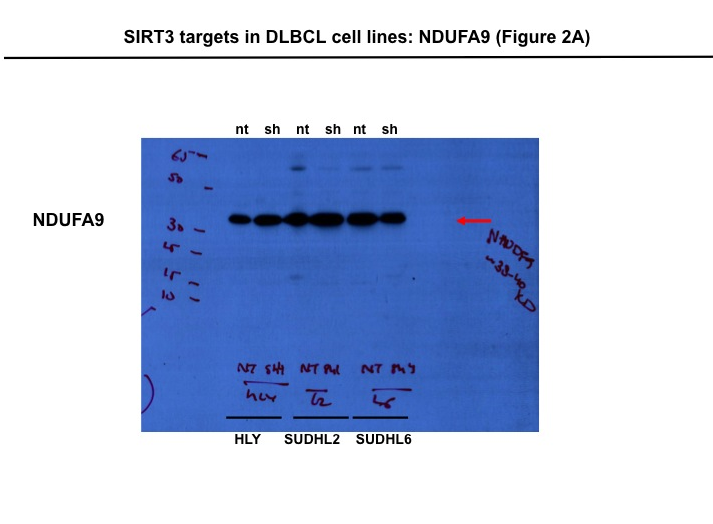

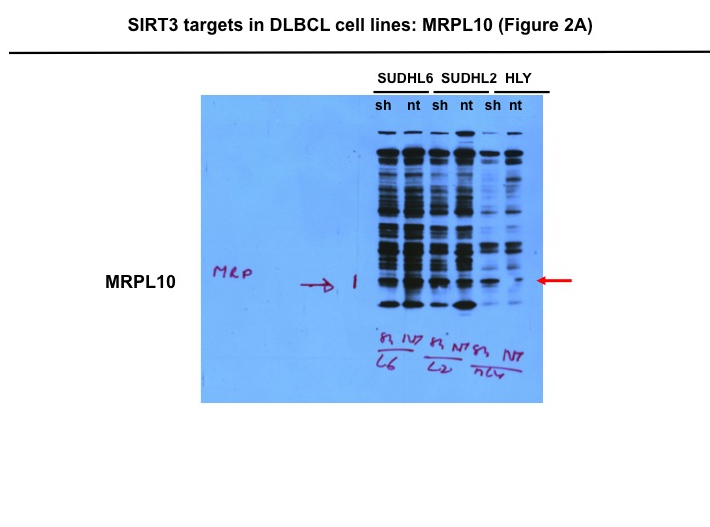

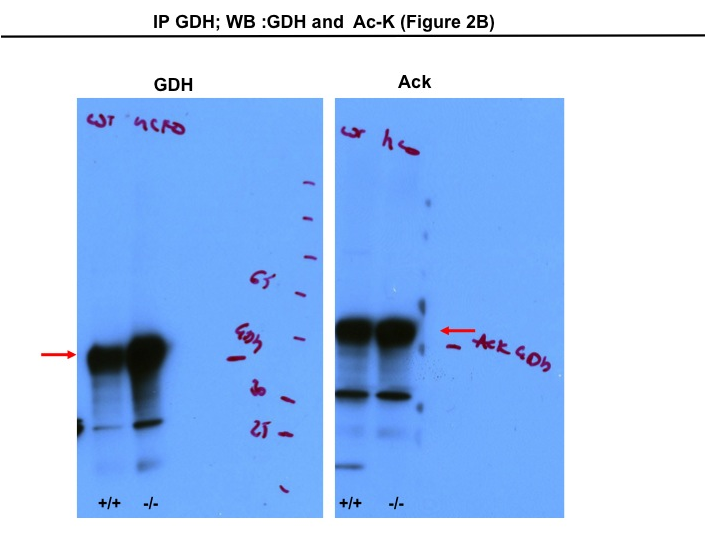

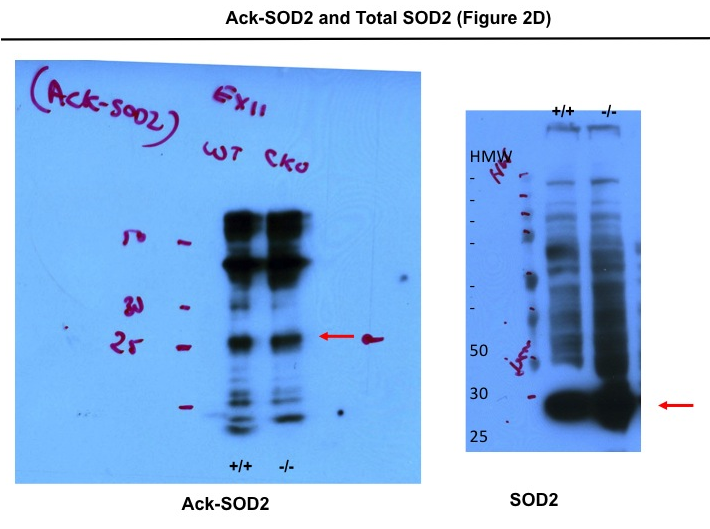

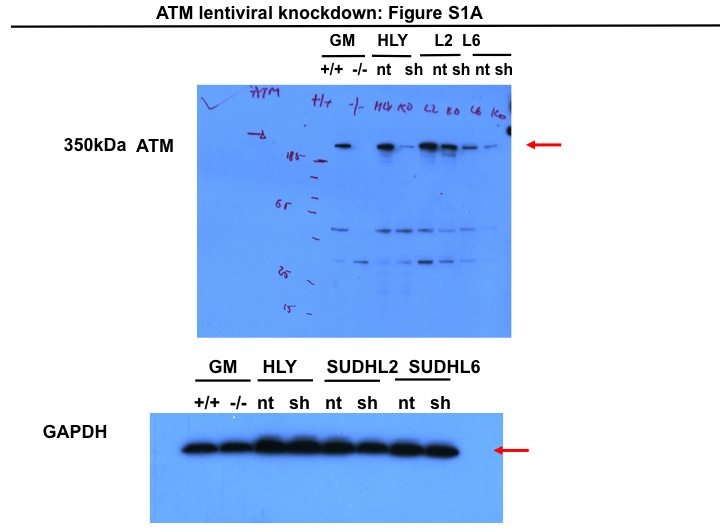

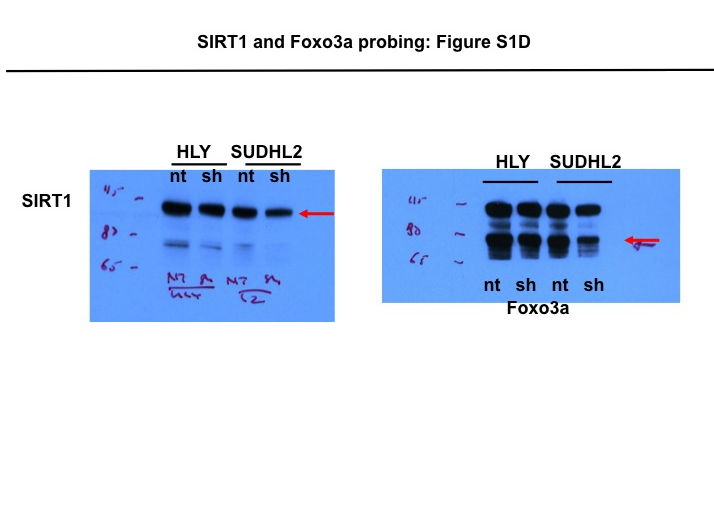

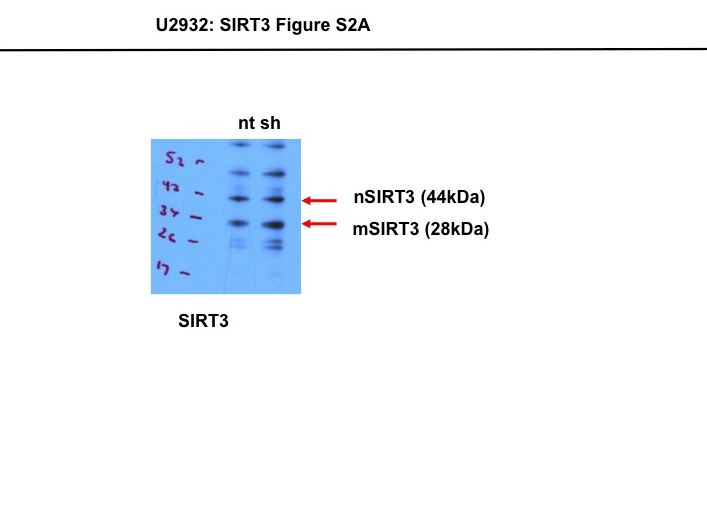

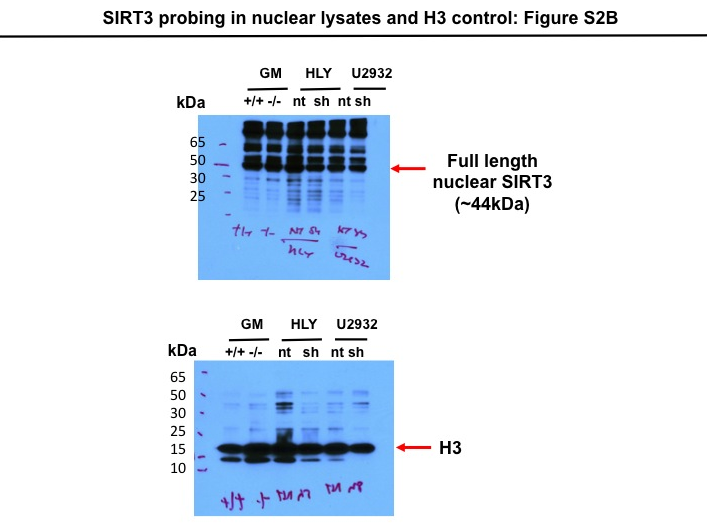

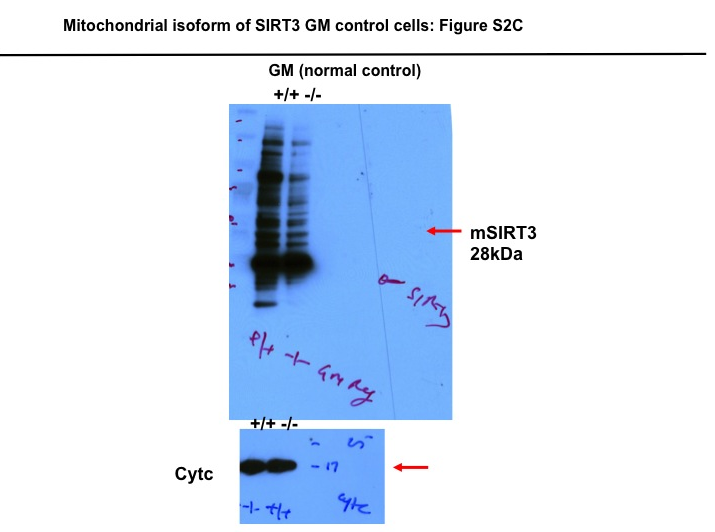

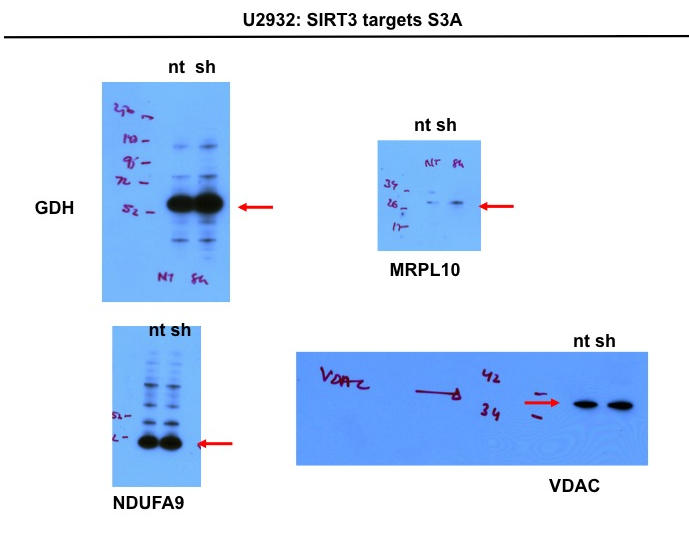

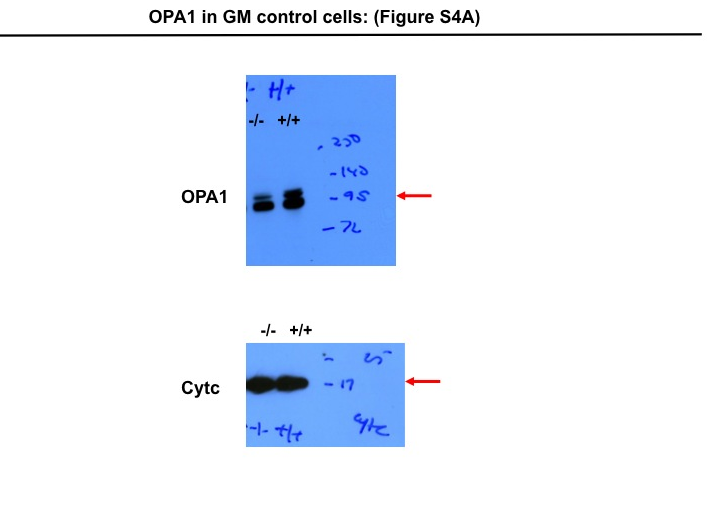

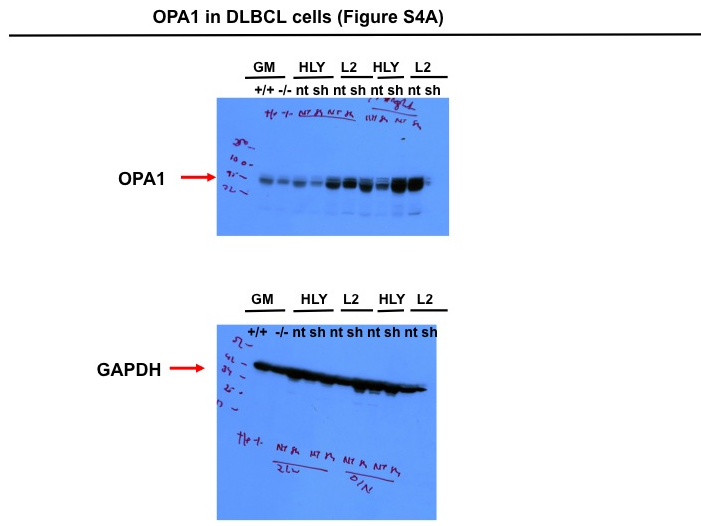

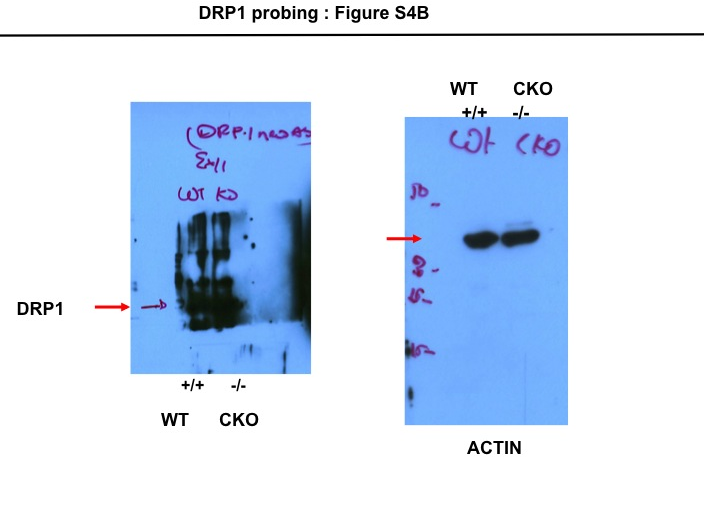

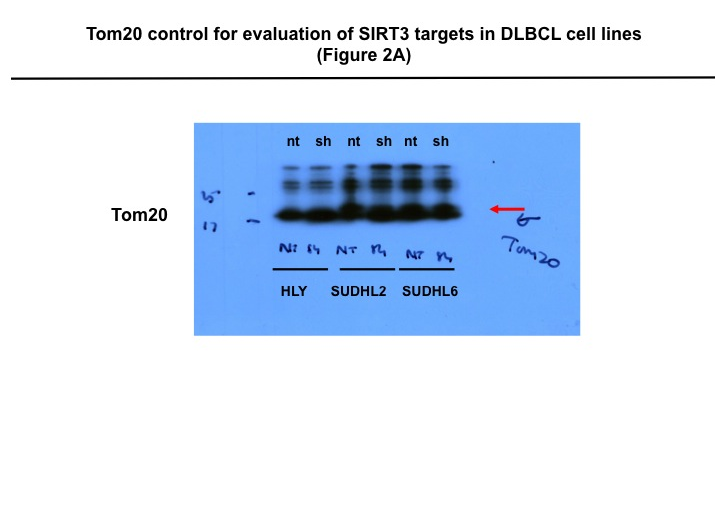

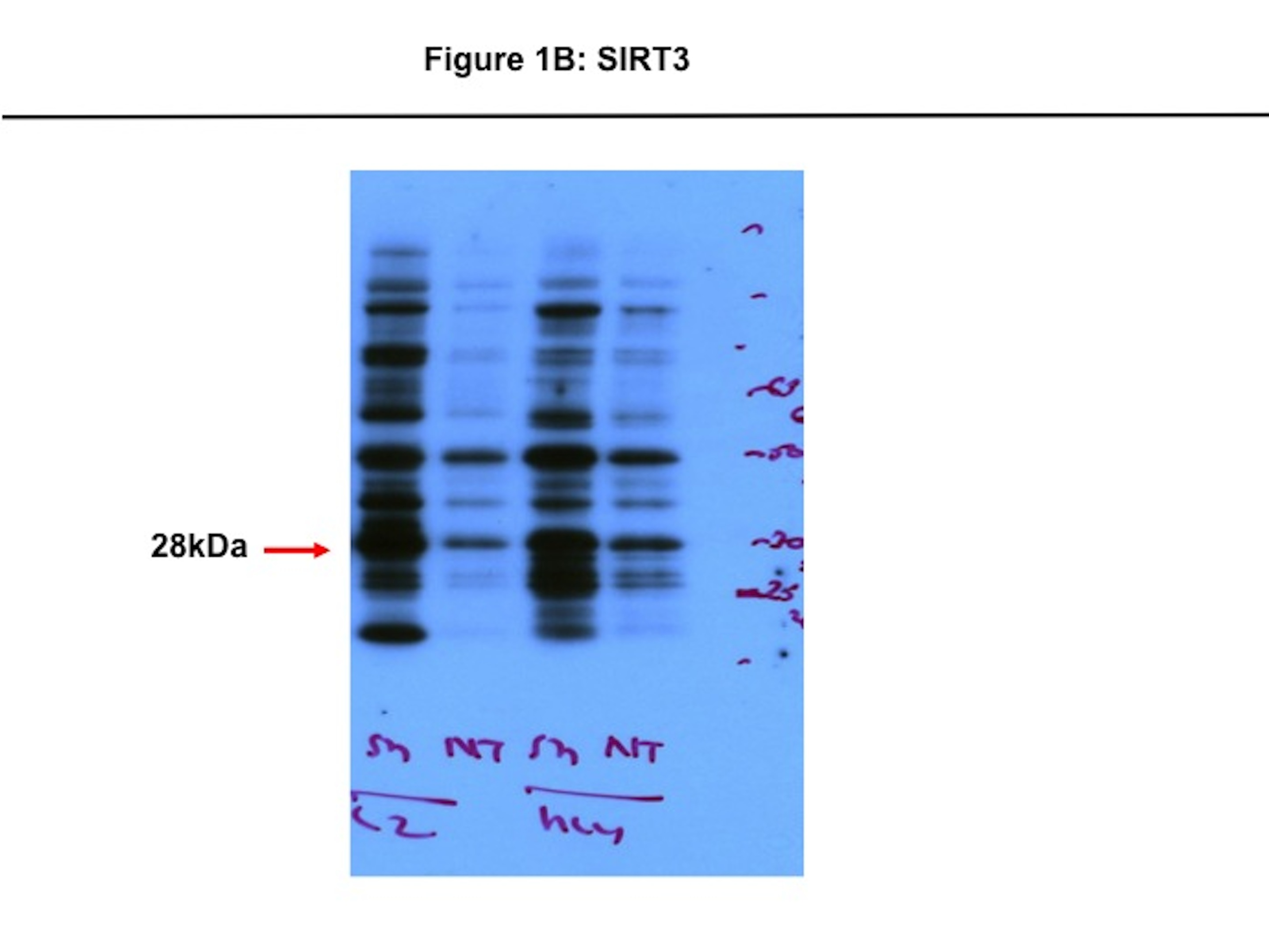

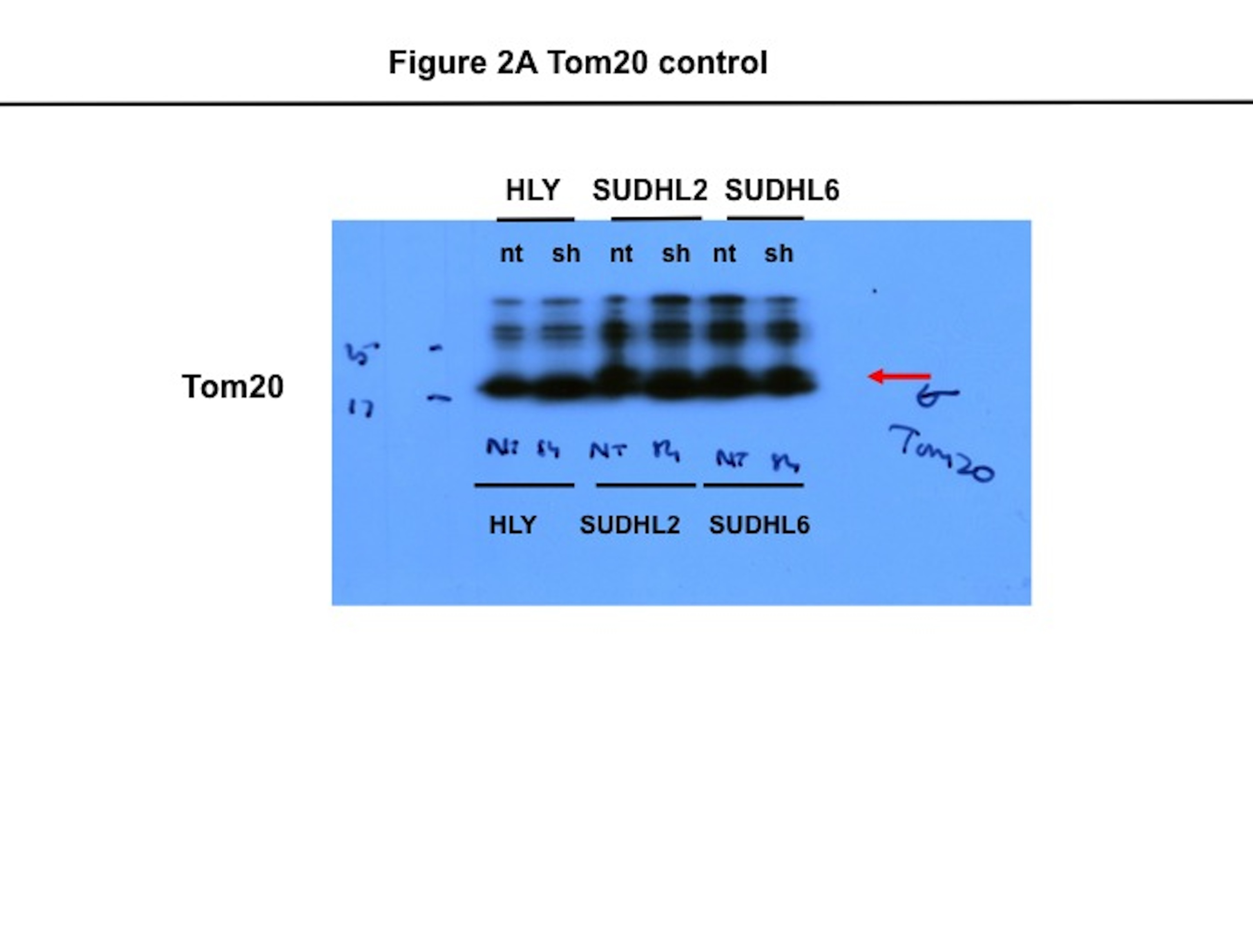

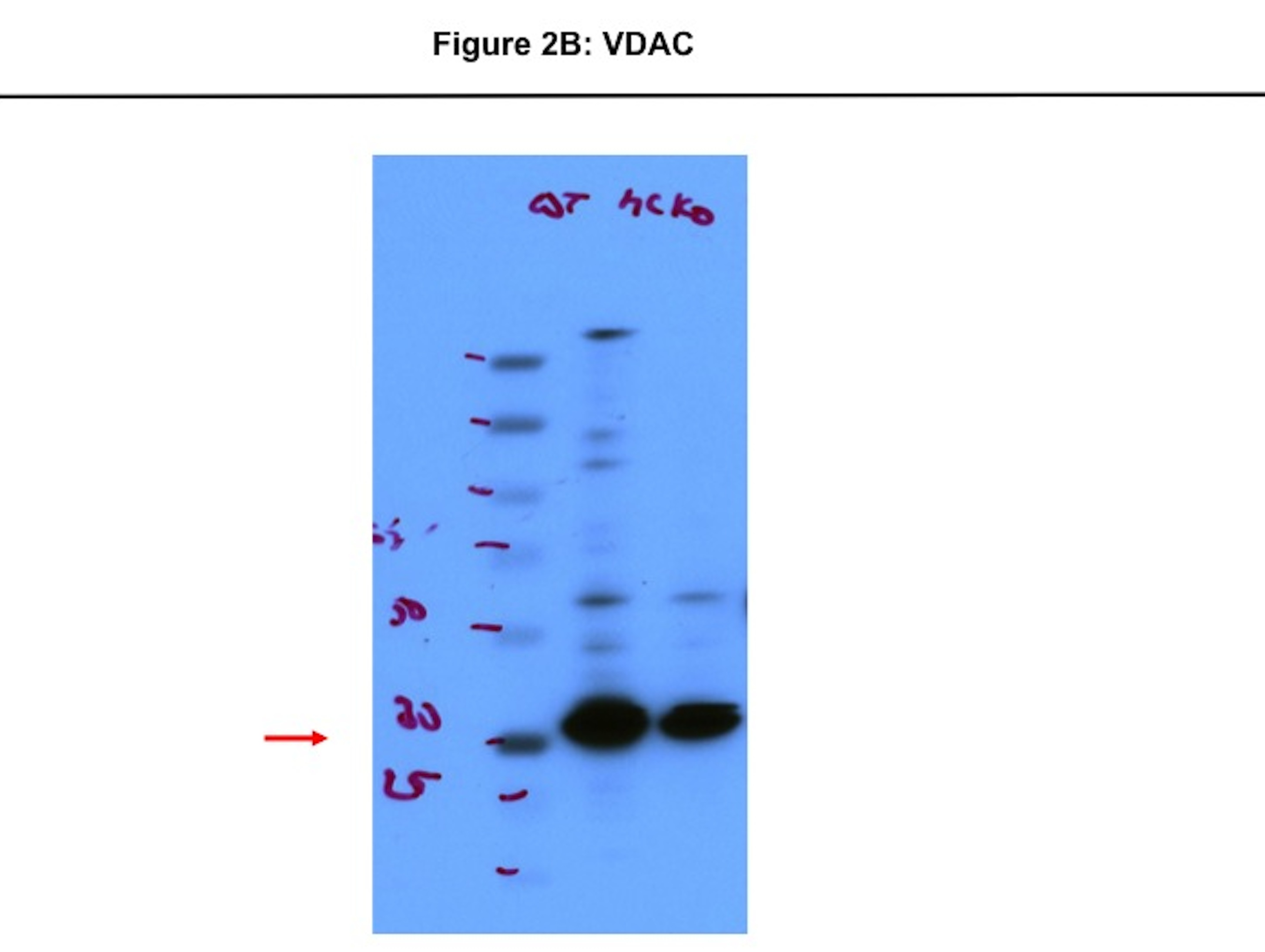

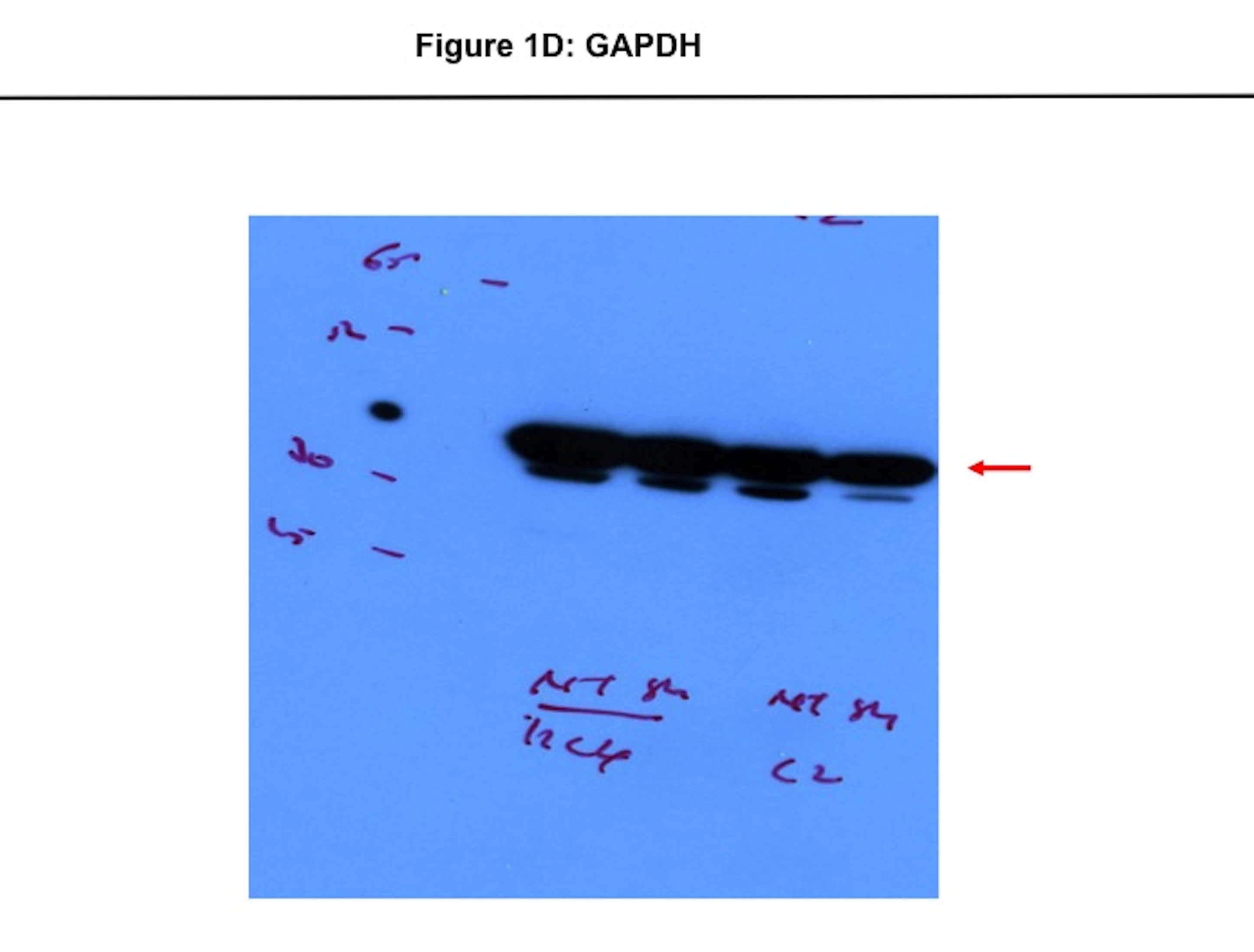

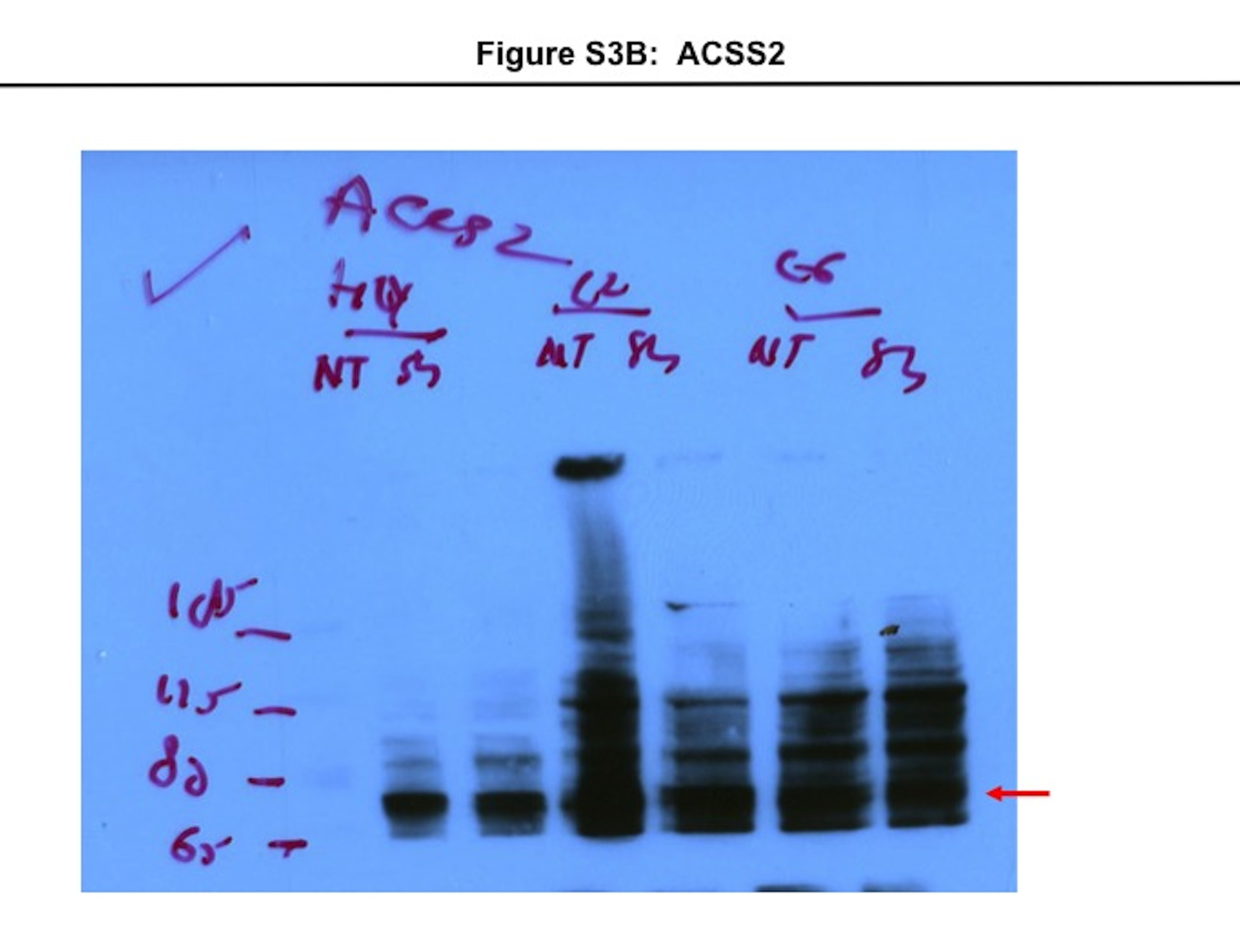

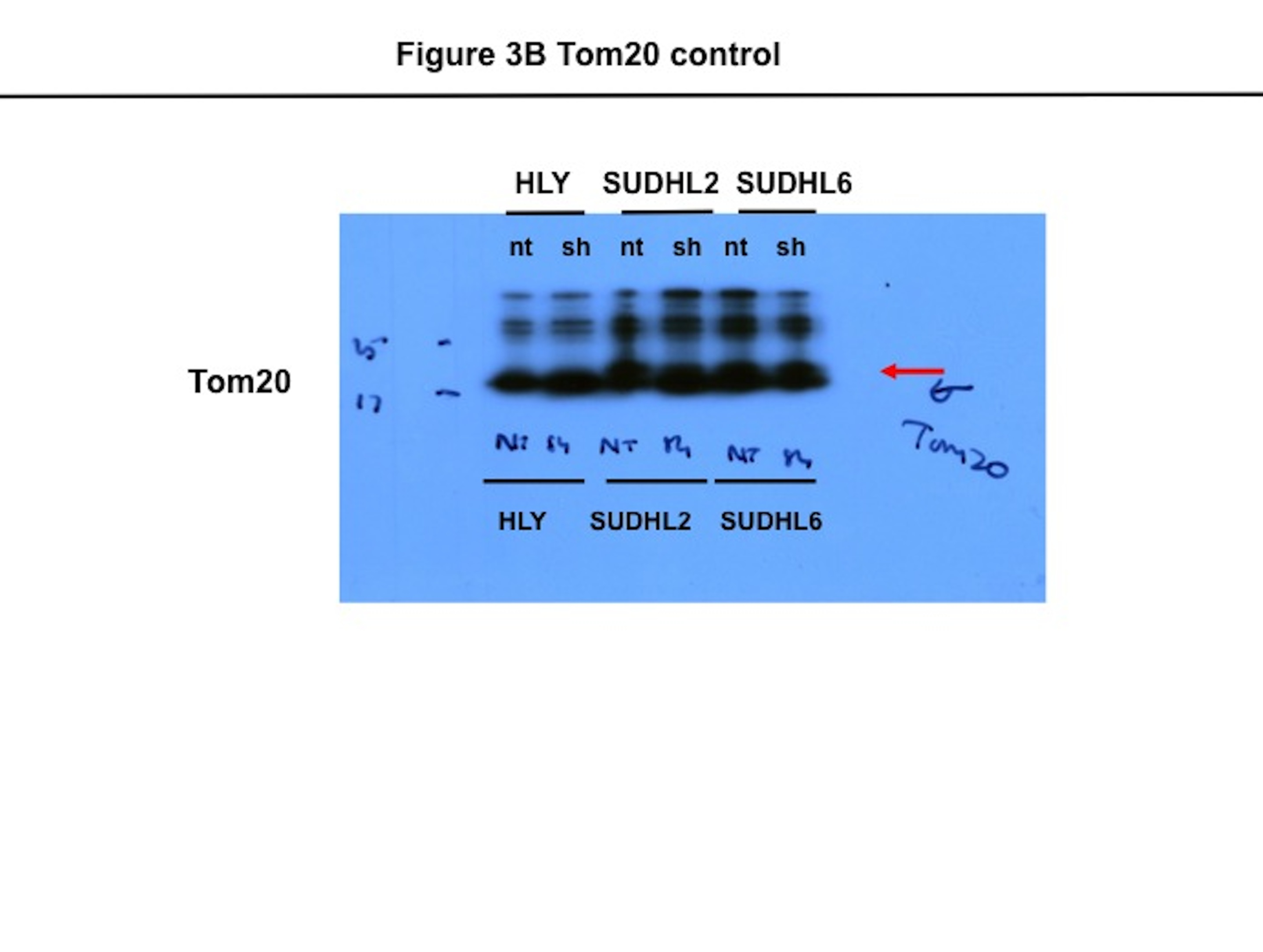

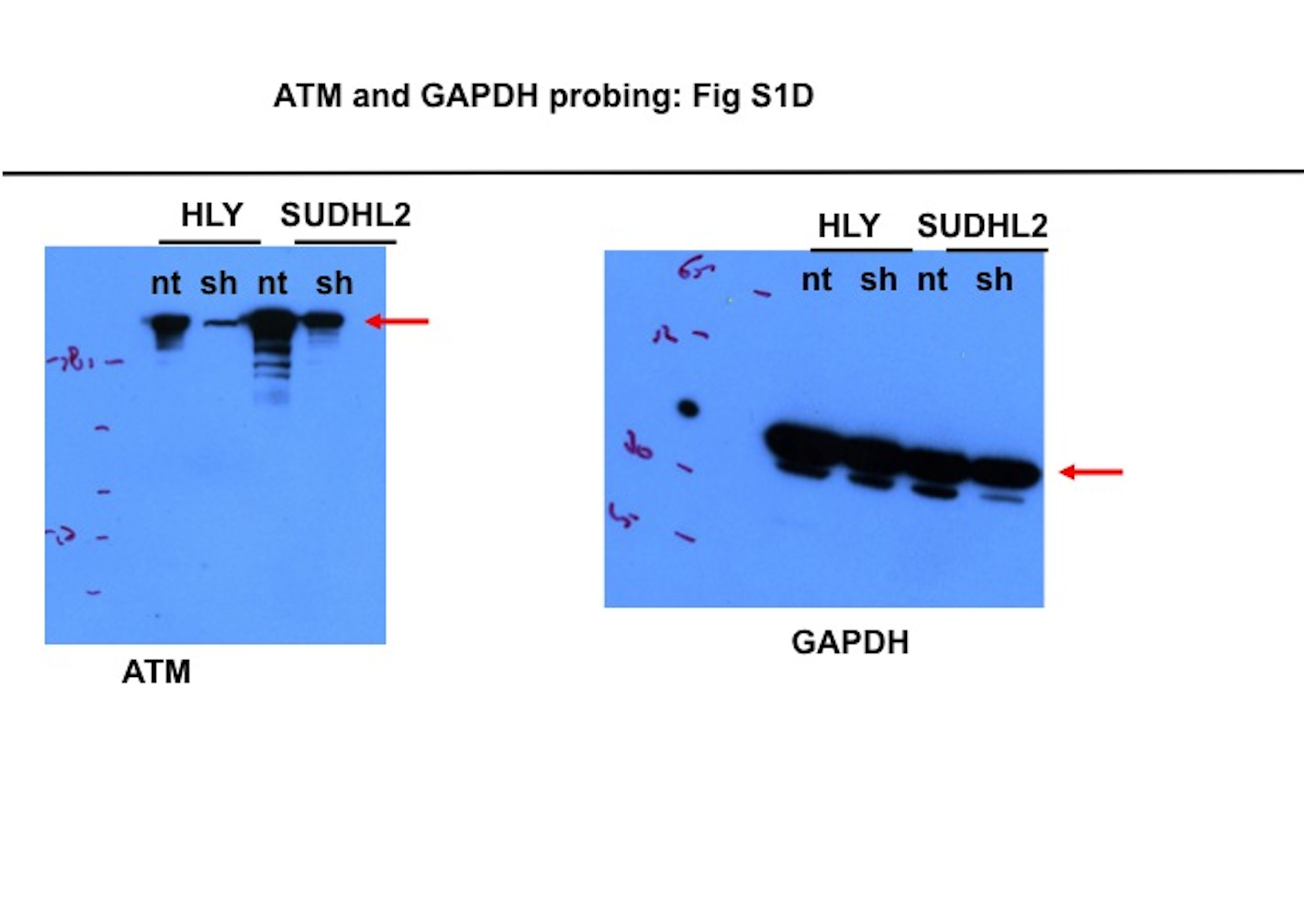

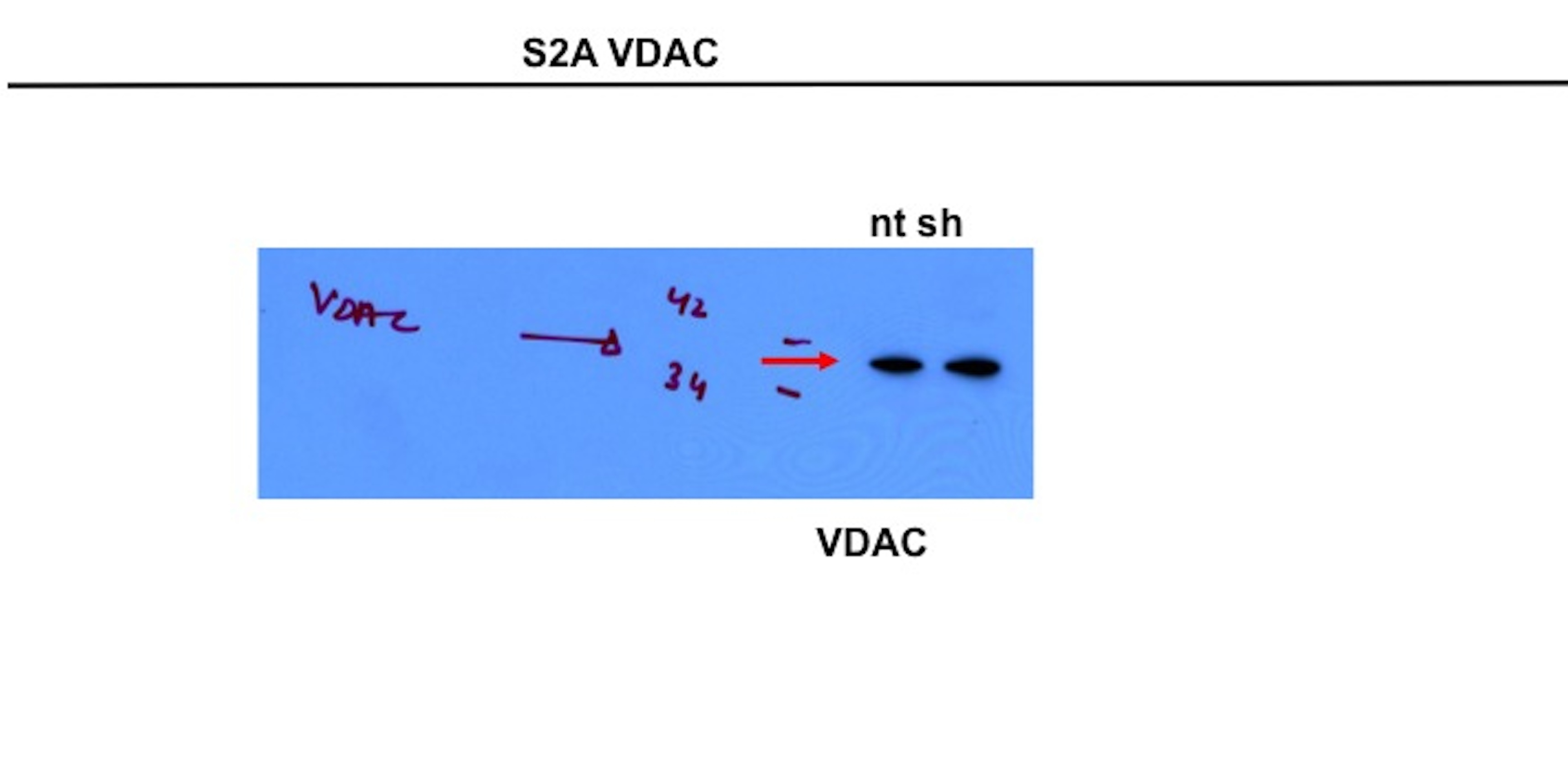

Supplement: Supplementary file 1 — Supplementary Information. [file 41598_2020_78193_MOESM1_ESM.docx]
